# Miocene *Cupressinoxylon* from Gökçeada (Imbros), Turkey with *Protophytobia* cambium mining and the study of ecological signals of wood anatomy

**DOI:** 10.7717/peerj.14212

**Published:** 2022-12-12

**Authors:** Dimitra Mantzouka, Ünal Akkemik, Yıldırım Güngör

**Affiliations:** 1 Senckenberg Natural History Collections Dresden, Königsbrücker Landstraße, Senckenberg Nature Research Society, Dresden, Germany; 2 Department of Forest Botany, Forestry Faculty, Bahçeköy-Sarıyer, Istanbul University-Cerrahpaşa, İstanbul, Turkey; 3 Department of Geology Engineering, Faculty of Engineering, Avcılar, İstanbul University-Cerrahpasa, İstanbul, Turkey

**Keywords:** Fossil wood, Ecological wood, Agromyzidae, Diptera, *Phytobia* Lioy, Plant-insect interactions, Endophytic phytophagy, Cambium miner, Miocene climate optimum, Miocene climate optimum north Aegean hotspot

## Abstract

**Premise:**

The recognition of the Miocene Climate Optimum (MCO) in terrestrial palaeoenvironments of the Eastern Mediterranean is restricted to Lesbos and Lemnos Islands, Greece. This area is significant for its wood microfossils. A recently-discovered fossil wood assemblage from Gökçeada (Imbros) Island, Turkey, including tree species similar to the Greek findings, is thought to have an early Miocene age. Here, we revise the age of the latter plant fossiliferous locality, re-evaluate the area for the study of MCO for the terrestrial palaeoecosystems of the Eastern Mediterranean and the nomenclature errors referring to the occurrence of fossil wood. We present the plant–insect–environment interactions using detailed anatomical descriptions, of an extinct conifer and its extinct cambium miner feeding traces observed in its secondary xylem.

**Methods:**

Three thin sections were prepared with standard palaeoxylotomical techniques from a small section of the silicified wood; the sections were observed under a light microscope. The anatomy of the conifer and its damage patterns were compared with those of extant and fossil Cupressaceae and Agromyzidae, respectively.

**Pivotal results:**

The common anatomical features of the studied wood specimen and *Hesperocyparis macrocarpa* (Hartw.) Bartel and a shared characteristic (the number of the cross-field pits – a feature we consider of diagnostic value) with *Xanthocyparis vietnamensis* Farjon & T.H. Nguyên led to its assignment to the *Hesperocyparis–Xanthocyparis–Callitropsis* clade. The detailed study of the wound scars and anatomical abnormalities, the anatomical–environmental associations, and structural–functional reactions follow the identification of the wood’s anatomy *sensu* Carlquist providing decisive results.

**Conclusions:**

Based on the distinctive characteristics presented, we identify our macrofossil as *Cupressinoxylon matromnense* Grambast, a stem or an extinct lineage of the *Hesperocyparis*–*Xanthocyparis vietnamensis–Callitropsis nootkatensis* clade with feeding traces of the fossil cambium miner of the genus *Protophytobia* Süss (Diptera: Agromyzidae), and anatomical damage and reaction tissue on adventitious shoots. The use of Protopinaceae and *Pinoxylon* F. H. Knowlton from the eastern Mediterranean are re–evaluated and corrections are provided. The age of the studied plant fossiliferous locality in Gökçeada is revised as middle Miocene, allowing the proposal of an eastern Mediterranean MCO hotspot, including Lesbos, Lemnos, and Gökçeada (Imbros) Islands.

## Introduction

The Aegean region of the Mediterranean basin has many plant fossil sites related to volcanism from the late Oligocene to the Miocene time. However, only Lesbos and Lemnos Islands are known for their early-middle Miocene petrified wood findings, providing a glimpse into Cenozoic vegetation and climate under coastal conditions: semihumid subtropical/humid warm temperate laurel forest palaeovegetation with deciduous elements (Cwa, according to the Kppen climate classification, more seasonal climate without pronounced dry season, Velitzelos, Bouchal & Denk, 2014). Lesbos Island, housing the well-known petrified forest in its western peninsula and additional plant fossiliferous localities with impressive upright and fallen silicified stems of early Miocene age (*e.g*., Süss & Velitzelos, 1993; Süss & Velitzelos, 1994a; Süss & Velitzelos, 1994b; Süss & Velitzelos, 1997, 1998, 1999, 2000, 2001, 2009, 2010; Süss, 1997, 2003; Mantzouka, 2016, 2018; Mantzouka et al., 2013, 2016, 2019; Mantzouka, Karakitsios & Sakala, 2017), represents a geoheritage monument internationally recognized by UNESCO. The material from Lesbos and Lemnos Islands is documented in Mantzouka et al. (2013, 2016, 2019), Mantzouka, Karakitsios & Sakala (2017), Velitzelos, Bouchal & Denk (2014), Mantzouka (2016, 2018), Velitzelos et al. (2019), and Iamandei et al. (2021). Anatomical studies of wood involving the microscopic sectioning of fossil wood structures during the last decade have revealed the occurrence of Cupressaceae similar to the wood in this study also from Lemnos Island (Mantzouka, 2016; Iamandei et al., 2021). Lesbos and Lemnos Islands continue to reveal new evidence of a rich fossil wood assemblage of angiosperms, including monocots and conifers that contain several type species for the early and middle Miocene (Table 1).

**Table 1:** Northeast Aegean Sea MCO hotspot.

Family	Fossil wood species identification	Type localities/Localities	Reference
**CONIFERS**			–
Cupressaceae	*Cupressinoxylon akdikii* Özgüven-Ertan	Lesbos and Lemnos Island	Iamandei et al. (2021)
Cupressaceae	*Cupressinoxylon matromnense* Grambast	Gökçeada Island, Çanakkale	This article
Cupressaceae	*Cupressinoxylon pliocenica* Akkemik	Gökçeada Island, Çanakkale	Akkemik (2020)
Cupressaceae	*Glyptostroboxylon microtraheidae* Süss & Velitzelos	Lesbos Island	Süss & Velitzelos (1997)
Cupressaceae	*Gl. rudolphii* Dolezych & van der Burgh	Lesbos and Lemnos Island	Iamandei et al. (2021)
Cupressaceae	*Gl. tenerum* (Kraus) Conwentz	Lemnos Island	Iamandei et al. (2021)
Cupressaceae	*Juniperoxylon acarcae* Akkemik	Lemnos Island	Iamandei et al. (2021)
Cupressaceae	*Taxodioxylon albertense* (Penhallow) Shimakura	Lesbos Island	Süss & Velitzelos (1997)
Cupressaceae	*T. gypsaceum* (Göppert) Kräusel	Lesbos Island, Lemnos Island, Gökçeada Island, Çanakkale	Süss & Velitzelos (1997), Akkemik (2020), Iamandei et al. (2021)
Cupressaceae	*T. lesbium* (Unger) Mantzouka & Sakala	Lesbos Island	Mantzouka, Karakitsios & Sakala (2017)
Cupressaceae	*T. megalonissum* Süss & Velitzelos	Lesbos Island	Süss & Velitzelos (1997)
Cupressaceae	*T. pseudoalbertense* M. Nishida & H. Nishida	Lesbos Island	Süss & Velitzelos (1997)
Cupressaceae	*T. taxodii* Gothan	Lemnos Island	Iamandei et al. (2021)
Cupressaceae	*Tetraclinoxylon velitzelosi* Süss	Lesbos Island, Lemnos Island	Süss (1997), Iamandei et al. (2021)
Cupressaceae	*Thujoxylon antissum* Süss & Velitzelos	Lesbos Island	Süss & Velitzelos (1998)
?Cupressaceae	*Thujoxylum peucinum* Unger (not *Thujoxylon* as stated in Süss & Velitzelos, 1998)	Lesbos Island	Unger (1847), Süss & Velitzelos (1998)
incertae sedis (Order: Ginkgoales)	*Ginkgoxylon lesboense* Süss	Lesbos Island	Süss & Velitzelos (1993)
incertae sedis (Order: Ginkgoales)	*G. diversicellulatum Süss*	Lesbos Island	Süss & Velitzelos (1993)
Pinaceae	*Chimairoidoxylon conspicuum* Süss & Velitzelos	Lesbos Island	Süss & Velitzelos (2001), family revised in this article
Pinaceae	*Ch. lesboense* Süss & Velitzelos	Lesbos Island	Süss & Velitzelos (1999), family revised in this article
Pinaceae	*Lesbosoxylon diversiradiatum Süss & Velitzelos*	Lesbos Island	Süss & Velitzelos (2010), family revised in this article
Pinaceae	*L. graciliradiatum* Süss & Velitzelos	Lesbos Island	Süss & Velitzelos (2010), family revised in this article
Pinaceae	*L. paradoxum* Süss & Velitzelos	Lesbos Island	Süss & Velitzelos (2010), family revised in this article
Pinaceae	*L. pseudoparadoxum* Süss & Velitzelos	Lesbos Island	Süss & Velitzelos (2010), family revised in this article
Pinaceae	*L. ventricosuratiatum Süss & Velitzelos*	Lesbos Island	Süss & Velitzelos (2010), family revised in this article
Pinaceae	*Pinuxylon* sp.	Gökçeada Island, Çanakkale	Güngör et al. (2019), D Mantzouka, Ü Akkemik & Y Güngör, 2022 (unpublished data) (genus revised in this article)
Pinaceae	*Pinuxylon halepensoides* van der Burgh	Lesbos and Lemnos Island	Iamandei et al. (2021)
Pinaceae	*Pinuxylon parenchymatosum* (Süss & Velitzelos) Mantzouka & Akkemik	Lemnos Island	Süss & Velitzelos (1993), D Mantzouka, Ü Akkemik & Y Güngör, 2022 (unpublished data) (genus revised in this article)
Pinaceae	*P. pineoides* (Kraus) Koeniguer	Lesbos and Lemnos Island	Iamandei et al. (2021)
Pinaceae	*Pinuxylon* sp. aff. *Pinus canariensis* C. Sm.	Lesbos Island	Iamandei et al. (2021)
?Pinaceae	*Cedroxylon* sp.	Lemnos Island	Berger (1953)
?Pinaceae	*Pityoxylon* sp.	Lesbos Island	Fliche (1898)
?Podocarpaceae	*?Podocarpoxylon articulatum* Süss & Velitzelos	Lesbos Island	Süss & Velitzelos (2000)
?Podocarpaceae	*?P.graciliradiatum* Süss & Velitzelos	Lesbos Island	Süss & Velitzelos (2000)
Taxaceae	*Taxaceoxylon biseriatum* Süss & Velitzelos	Lesbos Island	Süss & Velitzelos (1994b), Philippe et al. (2019)
?Taxaceae	*Taxoxylon priscum* Unger (syn. *Taxoxylum priscum* Unger)	Lesbos Island	Unger (1847, 1850)
**ANGIOSPERMS**			
Arecaceae	*Palmoxylon chamaeropsoides* Iamandei et Iamandei	Lesbos Island	Velitzelos et al. (2019)
Arecaceae	*Palmoxylon coryphoides* Ambwani & Mehrotra	Gökçeada Island, Çanakkale; Lesbos Island	Iamandei, Iamandei & Akkemik (2018), Velitzelos et al. (2019)
Arecaceae	*Palmoxylon daemonoropsoides* (Ung.) Kirchh.	Lesbos Island	Velitzelos et al. (2019)
Arecaceae	*Palmoxylon phoenicoides* Hofmann	Lesbos Island, Lemnos Island	Velitzelos et al. (2019)
Arecaceae	*Palmoxylon sabaloides* Greguss	Lesbos Island	Velitzelos et al. (2019)
Arecaceae	*Palmoxylon trachycarpoides* Iamandei et Iamandei	Lesbos Island, Lemnos Island	Velitzelos et al. (2019)
Arecaceae	*Rhizopalmoxylon daemonoropsoides* Iamandei et Iamandei	Lesbos Island	Velitzelos et al. (2019)
Arecaceae	*Rhizopalmoxylon phoenicoides* Iamandei et Iamandei	Lesbos Island, Lemnos Island	Velitzelos et al. (2019)
Arecaceae	*Palmoxylon coryphoides* Ambwani & Mehrotra	Gökçeada Island, Çanakkale	Iamandei, Iamandei & Akkemik (2018)
Betulaceae	*Alnoxylon* sp.	Gökçeada Island, Çanakkale	Güngör et al. (2019)
Betulaceae	*Eucarpinoxylon kayacikii* Akkemik	Gökçeada Island, Çanakkale	Akkemik (2021)
Betulaceae	*Ostryoxylon gokceadaense* Akkemik	Gökçeada Island, Çanakkale	Akkemik (2021)
Cornaceae	*Cornoxylon pappi* Berger	Lemnos Island	Berger (1953)
Ebenaceae	*Ebenoxylon* sp.	Lesbos Island	Fliche (1898), Süss (1987)
Fagaceae	*Fagoxylon radiatum* Süss	Gökçeada Island, Çanakkale	Akkemik (2021)
Fagaceae	*Quercoxylon* sp.	Lesbos Island	Mantzouka (2016)
Fagaceae	*Quercoxylon yaltirikii* Akkemik	Gökçeada Island, Çanakkale	Akkemik (2021)
incertae sedis	*Brongniartites graecus* Unger	Lesbos Island	Unger (1845, 1850)
Juglandaceae	*Juglandoxylon mediterraneum* (Unger) Kraus (syn. *Mirbellites lesbius* Unger, *Juglandinium mediterraneum* Unger)	Lesbos Island	Unger (1845, 1850), Kraus (1882a, 1882b), Fliche (1898), Dupéron (1988)
Lauraceae	*Cinnamomoxylon seemannianum* (Mädel) Gottwald	Lesbos Island	Mantzouka et al. (2016)
Lauraceae	*Cryptocaryoxylon grandoleaceum* Akkemik	Gökçeada Island, Çanakkale	Akkemik (2021)
Lauraceae	*C. lemnium* Mantzouka	Lemnos Island	Mantzouka (2018)
Lauraceae	*C. lesbium* Mantzouka	Lesbos Island	Mantzouka (2018)
Lauraceae	*Laurinoxylon* aff. *czechense* Prakash, Březinová & Bůžek	Lesbos Island	Mantzouka et al. (2016)
Lauraceae	*L*. aff. *diluviale* (Unger) Felix emend. Dupéron et al.	Lesbos Island	Mantzouka et al. (2016)
Lauraceae	*L*. cf. *daberi* Greguss	Lesbos Island	Mantzouka (2016)
Lauraceae	*L*. cf. *ehrendorferi* Berger	Lemnos Island	Mantzouka (2016)
Lauraceae	*L. ehrendorferi* Berger	Lemnos Island	Berger (1953)
Lauraceae	*L. litseoides* Süss	Gökçeada Island, Çanakkale	Akkemik (2021)
Platanaceae	*Platanoxylon catenatum* Süss & Müller-Stoll.	Gökçeada Island, Çanakkale	Akkemik (2021)

**Note:**

The identified fossil wood species (conifers and angiosperms, including palms) of the MCO hotspot of the Northeast Aegean Sea, eastern Mediterranean, localities and the related references.

**Figure 1:**
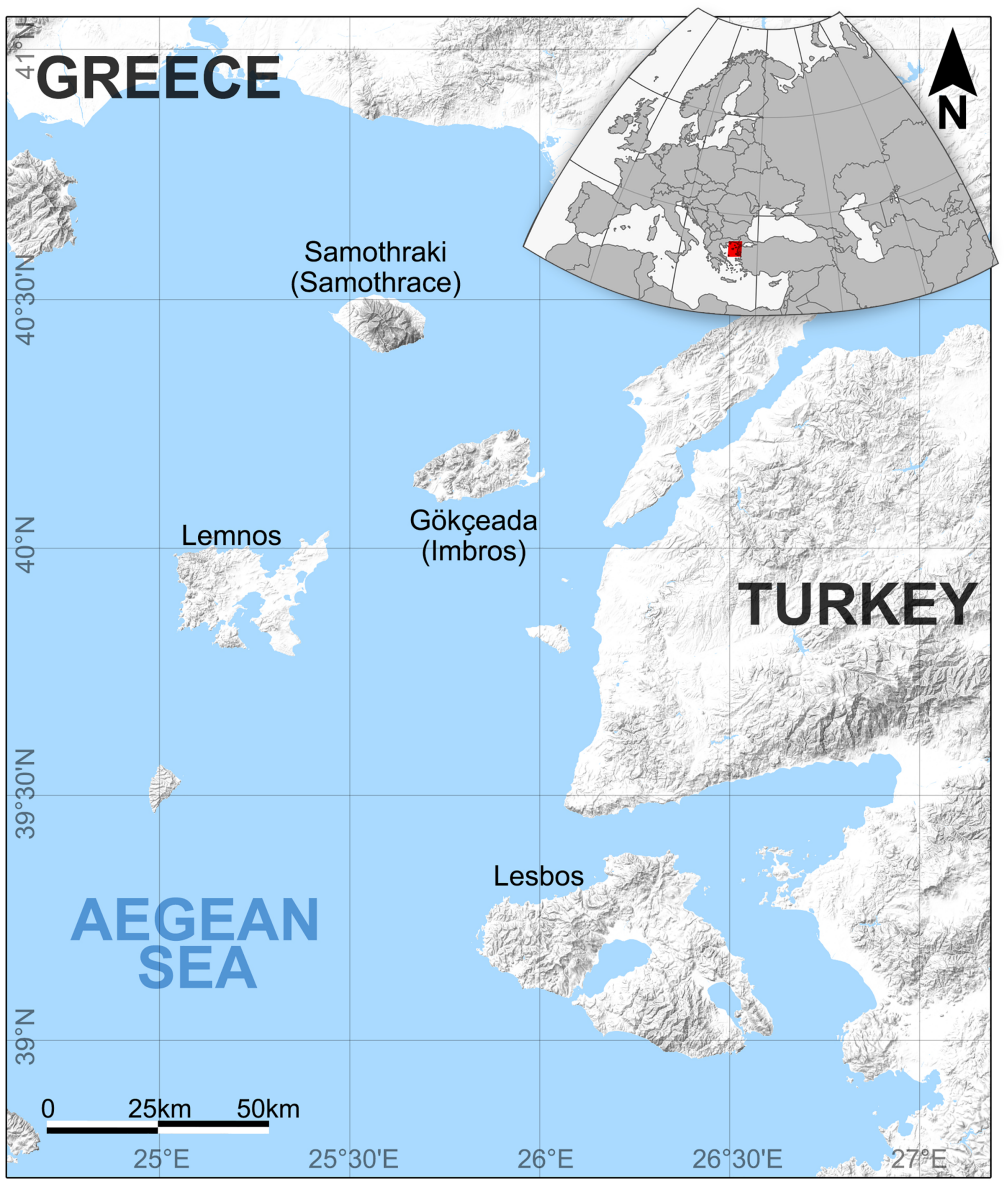
The location of Gökçeada island, Turkey and the Northeast Aegean Sea–eastern Mediterranean MCO hotspot. The location of the fossil wood site in Gökçeada island, Turkey along with the Northeast Aegean Sea–eastern Mediterranean MCO hotspot (including the Greek islands: Lesbos, Lemnos, and the Turkish: Gökçeada).

Gökçeada (Imbros) Island is located in the northern Aegean, 18 miles west of Çanakkale, north of Lesbos Island, northeast of Lemnos Island, and southeast of Samothraki (Samothrace) Island (Fig. 1). Geological similarities between Lemnos and Gökçeada Islands have been noted by De Launay (1898). In Gökçeada, a rich fossil wood assemblage recently has been found and identified by Iamandei, Iamandei & Akkemik (2018), Güngör et al. (2019), and Akkemik (2020, 2021). The first fossil wood findings of Gökçeada Island include conifers and angiosperms that include monocots (Table 1). This wood assemblage of Gökçeada indicates the presence of a subtropical, warm climate. The presence of palm trees (*Palmoxylon*), lauraceous woods (*Cryptocaryoxylon* and *Laurinoxylon*), taxodioid woods including swamp cypress (*Cupressinoxylon* and *Taxodioxylon*), alder (*Alnoxylon*), and plane trees (*Platanoxylon*) represent lower coastal and riparian conditions (see Acarca Bayam et al., 2018; Güngör et al., 2019; Akkemik, 2020, 2021). The co–occurrence of the latter woods with hornbeam, beech, and pine trees, indicates well–drained lowland and upland conditions (Güngör et al., 2019). Many other studies on the woody flora of early and early–middle Miocene have indicated the presence of a subtropical warm and humid climate in Turkey (*e.g*., Denk et al., 2017; Denk, Güner & Bouchal, 2019; Güner et al., 2017 on macrofossils; Akgün, Kayseri & Akkiraz, 2007; Akkiraz et al., 2011; Biltekin, 2017 on microfossils, and Acarca Bayam et al., 2018; Akkemik, 2020, 2021; Akkemik, Acarca & Hatipoğlu, 2017; Akkemik, Akkılıç & Güngör, 2019; Akkemik, Köse & Poole, 2005; Akkemik, Mantzouka & Kıran Yıldırım, 2020; Akkemik & Sakınç, 2013; Akkemik et al., 2009, 2016, 2019 on petrified woods).

New fossil wood material occurences accompanied with microscopic observations have contributed to the knowledge of the woody flora. The petrified woods previously described from Gökçeada belong to the same fossiliferous outcrop hosting the fossil wood described in this article, originating from various volcanic eruptions or different depositional environments between major volcanic events. The fossiliferous locality has been exposed on the surface of a slope along the southeastern aspect of the Island due to erosion and weathering. As a result, there are occurences of silicified and charcoalified remnants of fossil trees along the shore, but their retrieval can be very difficult due to an inhospitable topography.

Identification of the greenhouse/warming event called the Miocene Climatic Optimum (MCO) principally originates from marine data (Zachos et al., 2001; Zachos, Dickens & Zeebe, 2008; Shevenell, Kennett & Lea, 2004; Westerhold et al., 2020). Consequently, evidence from the terrestrial responses from Central Europe (*e.g*., Mosbrugger, Utescher & Dilcher, 2005), with the addition of palaeobotanical and mammal/hypsodonty data (Saarinen, Mantzouka & Sakala, 2020) from the eastern Mediterranean (Greece and Turkey) during increased temperatures through the early and middle Miocene, could improve our knowledge significantly and provide a combined account of the biotic events that occurred during this crucial period. Additionally, the establishment of an eastern Mediterranean MCO hotspot, including Lesbos, Lemnos, and the Gökçeada (Imbros) Islands may be supported by the detection of palaeoclimatic signals through the wood anatomical traits of xylem (Pandey, 2021), as well as the palaeocommunities *sensu* Watkins, Berry & Boucot (1973); metacommunities *sensu* Leibold et al. (2004) and Leibold, Rudolph & Blanchet (2020) or palaeo–metacommunities following the example of Blanco et al. (2018) and García-Girón et al. (2021).

In the context of this discussion, the goal of the present study is to identify the microscopic thin sections of new fossil woods from Gökçeada assigned to *Cupressinoxylon* to provide information about the possible causes of the anatomically observed wound scars as well as an additional contribution and correction to the woody flora of the Island that has been assigned to a middle Miocene age. We believe this contribution is of high importance for a better understanding of the Miocene Climate Optimum interval that has been essential for the history of life on earth.

## Materials and Methods

### Sampling and analysis

The fossil wood was collected by Yıldırım Güngör from the southeastern side of Gökçeada Island, which includes fossil wood localities along small valleys and the coastline (Fig. 1). The results of the first collection (a total of 16 samples) revealed 11 different fossil species from the area (Akkemik, 2020, 2021; Güngör et al., 2019). Our new specimen comes from the same fossiliferous area. The diversity of species of angiosperms including palms and conifers found mixed in the same slope exposure was determined to be of parautochthonous origin (Güngör et al., 2019). This hypothesis is supported by fossil material which was deposited along the coast, likely after a short move. The extremities of the logs were not abraded or rounded, as they were transported by a lahar from the upper, mid, and lower parts of the surrounding valleys.

The length of the specimen is approximately 5 cm and its diameter is 2 cm. The specimen was silicified and may belong to a branch rather than a main trunk. Slide preparations (transverse, tangential, and radial) were made for anatomical study and qualitative and quantitative analyses were performed with a Leica^©^ DM 2500 light microscope, equipped with a Leica^©^ digital camera (DFC 295). We used the International Association of Wood Anatomists (IAWA) terminology for softwood (IAWA Committee, 2004) along with the system of Esteban et al. (2004). The classification of the fossil conifer woods follows the modern systematics presented by Christenhusz et al. (2011). Original preparations from Schönfeld’s fossil collection (Specimen numbers A4, A10, E11, H41, H44, H58, H65, H69, H70.1, H70.2, H74, R14.3), first studied by Kräusel & Schönfeld (1924), along with slides of extant material belonging to Schönfeld’s recent collection (n^o^: 29) housed in the Senckenberg Natural History Collections, Dresden, Germany (abbreviation: SNSD), were studied for comparison purposes. This material was studied with a Leica^©^ DM 5500 light microscope, equipped with a Leica^©^ digital camera (DFC 480).

The material from Gökçeada studied in this work has been permanently accessioned into the official collection reposited at Istanbul University–Cerrahpaşa, Forestry Faculty, Department of Forest Botany under the accession number GOK17. The Ministry of Agriculture and Forestry General Directorate of Nature Conservation and National Parks approved this study (E-21264211-288.04-744472).

### Geological setting

**Figure 2:**
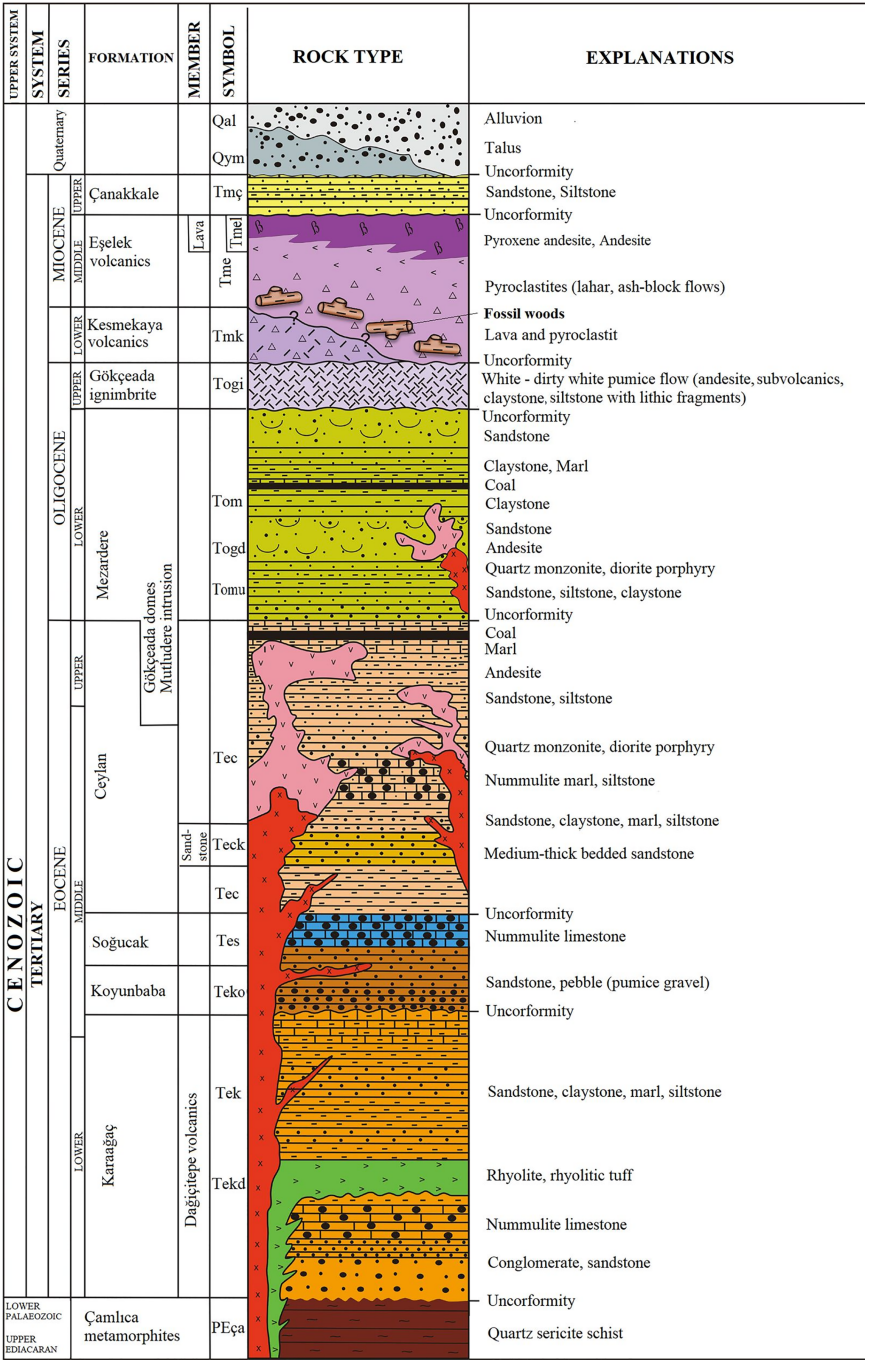
Stratigraphic column of the studied fossiliferous area (modified from Sarı et al., 2015). Image license: CC-BY-NC-ND 4.0.

The fossil wood material of Gökçeada (Imbros) Island was found in the southeastern part of the Island. The age of the fossil wood was thought to be early Miocene, the same as the Kesmekaya volcanics (Güngör et al., 2019). A detailed reappraisal of the literature (Akartuna, 1950; Sarı et al., 2015; Şen et al., 2020) showed that the fossil woods belonging to the Eşelek Pyroclastics Formation is regarded as middle Miocene age. The “Eşelek Volcanic Rocks” were named and mapped by Sarı et al. (2015). They are situated along the eastern aspect of the Island, and consist of lahars and blocky ash flows (debris flows and pyroclastics) (Sarı et al., 2015; Şen et al., 2020). Sarı et al. (2015: 12) reported the existence of peat deposits interbedded within the tuffs. The age of the Eşelek volcanic deposits is considered to be middle Miocene because it underlies unconformably the upper Miocene Çanakkale Formation and it overlies the Mezardere Formation and Gökçeada Ignibrite, both of which have a lower Oligocene age, and the Kesmekaya volcanic strata (Fig. 2). Kesmekaya volcanics overlie Gökçeada Ignimbrite and its regional correlation with the early Miocene formations of the Biga Peninsula is accepted (Sarı et al., 2015). The age of the Eşelek volcanics must be early–middle Miocene because this formation was unconformably overlaid by the Canakkale Formation, which is thought to have a late Miocene (Pontian) age (Akartuna & Atan, 1978). The age of the latter formation comes from the Biga Peninsula and is regarded as Late Miocene by Atabey, Ilgar & Saltık (2004) and currently is generally accepted (Fig. 2). We support the attribution of Eşelek volcanic formation as early-middle Miocene, acknowledging that more palaeontological and radiometric evidence is needed. According to the geochemical study by Şen et al. (2020: 94), the andesitic Eşelek volcanics have a tholeiitic composition and are *“products of post–collisional magmas retaining subduction signatures”*.

The vicinity of the volcanic fossiliferous locality of Gökçeada both at Lemnos and Lesbos Islands and at Galatian Province, with the related age and composition of the volcanism and the fossilization type of the material, can give a portrait of this area during the Miocene Epoch when volcanic activity was present along with the mixed forests of conifer and broad–leaved trees were the dominant vegetation in this basin.

**Figure 3:**
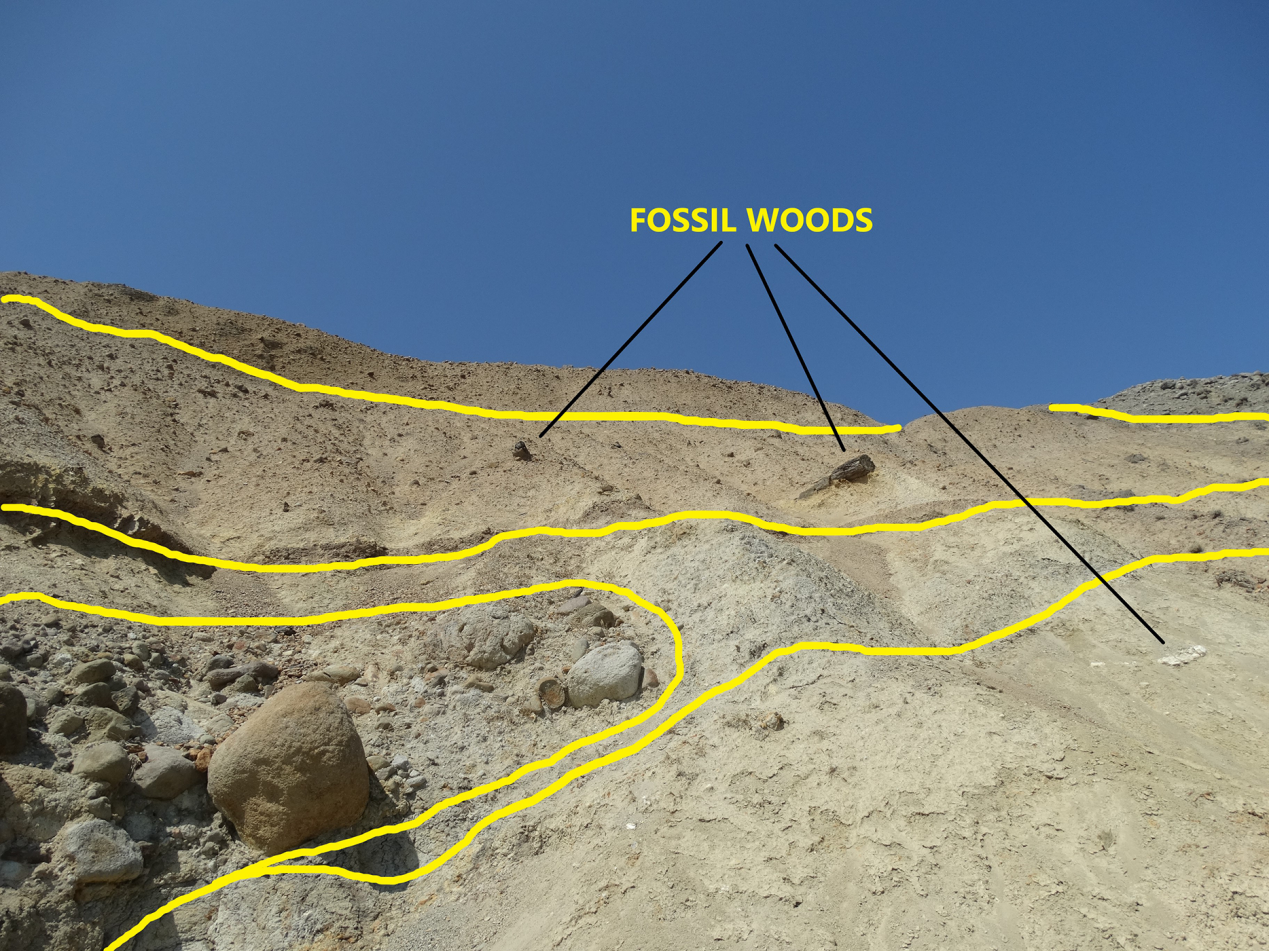
The outcrop of the plant fossiliferous locality in Gökçeada, showing fossil woods of different fossilization types occurring in different depositional units.

Field observations regarding the position of the woods along with their different fossilization types of charcoalified, silicified and calcified, provided identification of different volcanic events (represented by possibly up to four different depositional units that presently are accompanied by wood fragments with a variety of fossilization types, as shown in Fig. 3). Based on the study of Allen (2001), altered depositional units with related wood discoveries can belong to the same or different volcanic eruption or to different depositional environments between major eruptive events, as in the Lesbos case (Pe-Piper et al., 2019). According to the later authors, in the Lesbos case study, the volcanic activity and pyroclastic deposition lasted at least 4 million years, ranging from 20.5–21 Ma (Bali Alonia) to 16.0 ± 0.5 Ma at Nissiopi Islet.

## Results and discussion

**Systematic paleontology**

Subclass PINIDAE Cronquist, Takhtajan et Zimmermann, 1966 (=conifers)

Order PINALES Gorozhankin, 1904

Family CUPRESSACEAE Gray 1821 *sensu* Farjon 2005

Genus CUPRESSINOXYLON Göppert, 1850 emend. Dolezych, 2005

***Cupressinoxylon matromnense* Grambast, 1952**

**Figure 4:**
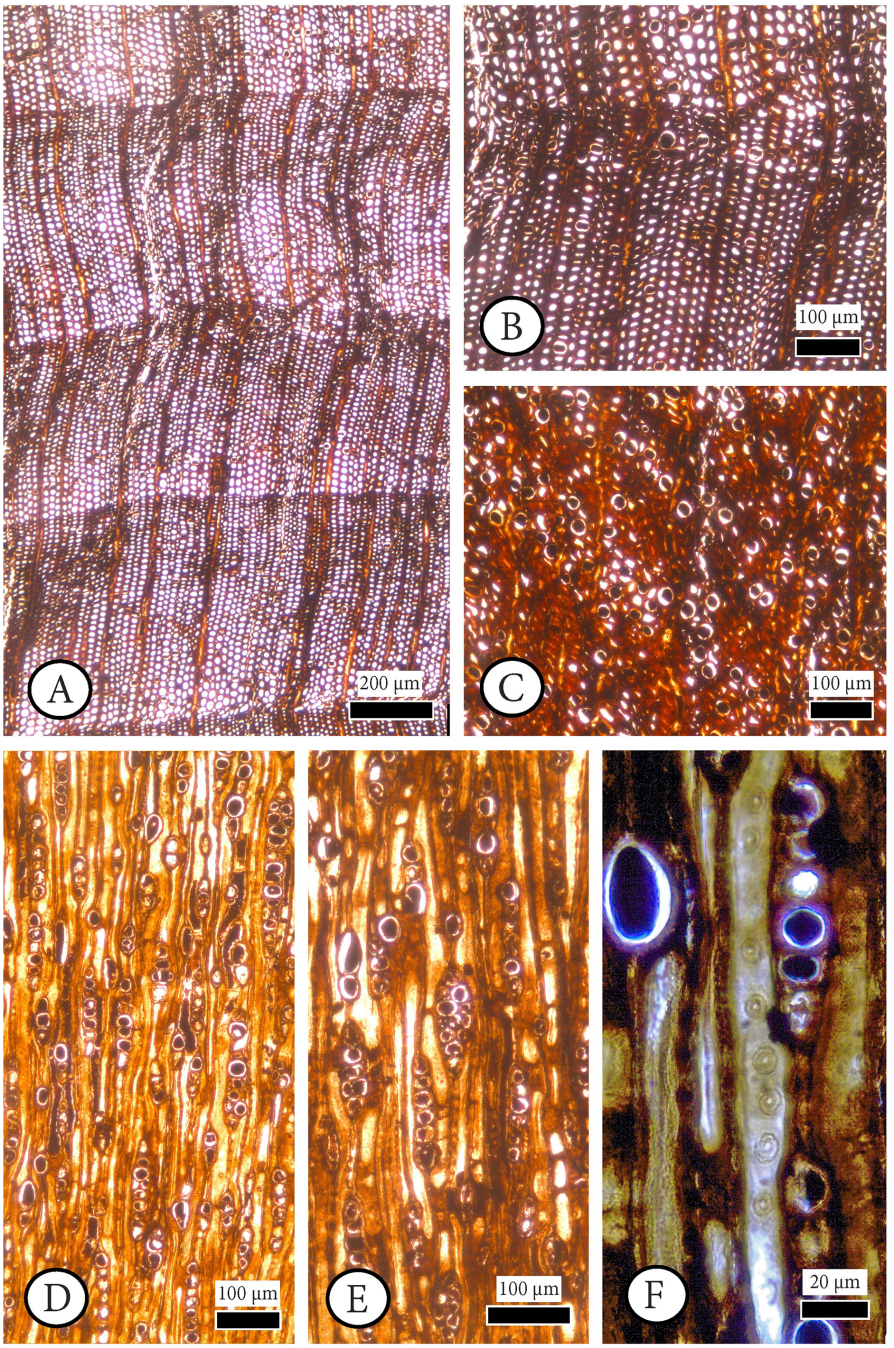
*Cupressinoxylon matromnense* Grambast from Gökçeada, Turkey. Material: GOK17. (A, B) Growth ring boundaries distinct; transition from early- to latewood gradual. Axial parenchyma diffuse and marginal. Axial tracheids mostly circular. (C) Growth ring boundaries indistinct in narrow rings. Axial parenchyma diffuse and marginal. Axial tracheids circular. (D) Rays short, mostly uniseriate, sometimes partly biseriate. (E) Rays short, mostly uniseriate, partly biseriate and rarely triseriate. (F) Bordered pits present on tangential walls of the axial tracheids. (A–C): transverse section, (D–F): tangential section.

**Figure 5:**
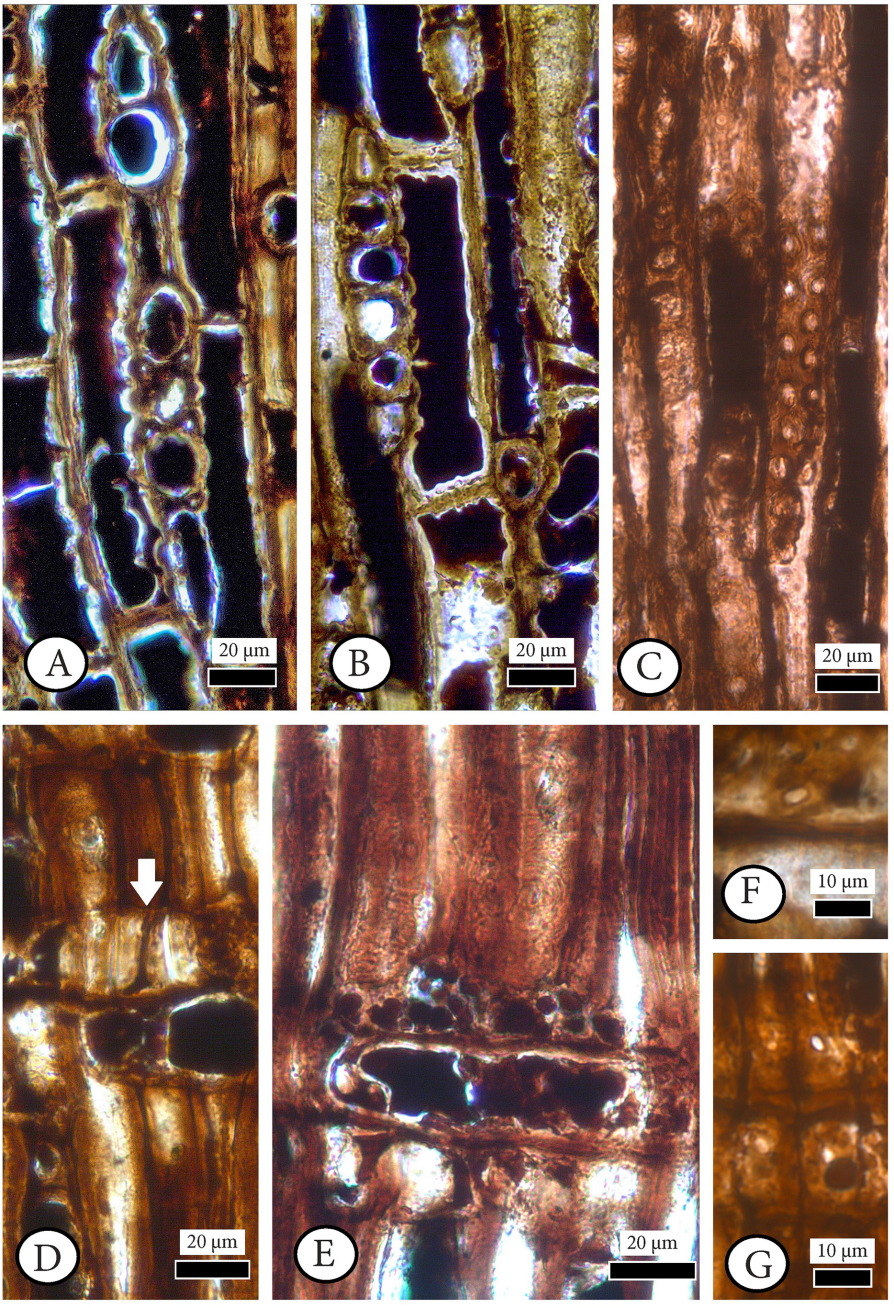
*Cupressinoxylon matromnense* Grambast from Gökçeada, Turkey. Material: GOK17. (A, B) Transversal end walls of axial parenchyma mostly smooth and sometimes slightly nodular. Dark content very common in axial parenchyma. (C) Bordered pits on radial walls of axial tracheids predominantly uniseriate, sometimes partly biseriate. (D) Indentures present. Ray parenchyma with dark content commonly. (E) Rays heterogenous. Ray tracheids (or degenerated cells) present. (F, G) Cross-field pits cupressoid, with 1–4 pits per cross field (A and B): tangential section, (C–G): radial section.

**Figures 4, 5, Table S1.**

**Material.** GOK17 (specimen and three slides)

**Repository.** Istanbul University–Cerrahpaşa, Forestry Faculty, Department of Forest Botany

**Locality.** East of Eşelek Village–Gökçeada Island, adjacent the coastline.

**Age.** Middle Miocene.

**Wood description.** Description of the wood was made on the three thin sections of a silicified wood with about 5 cm in diameter and 2 cm in length. It is very likely branch wood.

**Transverse section.** Growth ring boundaries distinct; transition from early–to latewood gradual (Figs. 4A, 4B) and indistinct in narrow rings (Fig. 4C). No resin canals present. Different types of scars observed near to latewood zone, and at the beginning of earlywood of some growth–rings. Latewood zone not clear and a couple of flattened latewood tracheids can be seen. Some incompletely developed rings observed. Tracheids mostly circular (Figs. 4A–4C). Tracheids are generally small; tangential and radial diameters of tracheids are 19 (10–27) µm and 17 (5–28) µm in earlywood, respectively. They are 12 (6–19) µm and 5 (2–7) µm in latewood. Double wall thickness of tracheid cells is 7 (3–9) in earlywood and 6 (4–9) µm in latewood. In earlywood, small intercellular spaces are present. Axial parenchyma is common not only in the transition zone from early–to latewood and in the latewood zone but also through the growth ring and in earlywood. The axial parenchyma is diffuse and situated also in the latewood zone (Figs. 4A–4C). Axial parenchyma diameter in the transverse section is 20 (13–44) µm.

**Tangential section.** Rays mostly uniseriate, sometimes partly biseriate, and rarely triseriate (Figs. 4D, 4E). Ray height 1–24 cells, and 101 (26–251) µm. Diameters of ray cells irregular, ray width 22 (range from 10 to 29) µm in uniseriate ones and 30 (range from 20 to 46) µm in bi–triseriate ones (Fig. 4E). Number of rays per mm^2^ 30–50. Diameter in the widest part of axial parenchyma in tangential section 29 (18–39) µm. Bordered pits present on tangential walls of the axial tracheids (Fig. 4F). Diameter of tracheid pittings in tangential walls is 11 (8–14) µm >10 µm. Transversal end walls of axial parenchyma mostly smooth and sometimes slightly nodular. Dark content very common in axial parenchyma (Figs. 5A, 5B).

**Radial section.** Transverse end walls of axial parenchyma in radial section are smooth. Lumen diameter in the widest part of axial parenchyma in radial section 28 (15–30) µm, transverse end wall thickness in radial section 6 (3–8) µm. Bordered pits on radial walls of axial tracheids predominantly uniseriate, sometimes partly biseriate (Fig. 5C). Indentures present (Fig. 5D). Rays homogenous in general, and ray tracheids (or degenerated cells) sometimes present (Fig. 5E). Ray parenchyma with smooth end walls present and with unpitted horizontal and tangential walls (Figs. 5D, 5E). Cross–field pits cupressoid, with 1–4 (very rarely 5) pits per cross field and pit’s diameter: 5 (4–6) µm (Figs. 5F, 5G). Ray parenchyma has dark content commonly (Figs. 5D, 5E). Crassulae absent. No crystals present. Diameter of tracheid pitting in radial walls is 10 (9–12) µm, and the diameter of their pores 3 (2–4) µm.

**Discussion.** The examined wood has rounded tracheids, and the axial parenchyma transverse end walls are mostly smooth (and sometimes slightly nodular), displaying cupressoid with 1–4 (very rarely–5) pits per cross–field and the average height of procumbent ray cells less than 25 μm. The ray parenchyma has a smooth end and horizontal walls. These characteristics could correspond to *Tetraclinoxylon* Grambast as described in Vaudois & Privé (1971). A more detailed study of the characteristics of this genus reveals that *Tetraclinoxylon* includes woods with narrow cross–field pits apertures, absence of indentures, and only smooth axial parenchyma walls.

Taking into account the key of fossil Cupressaceae by Vaudois & Privé (1971), we determined that our fossil as similar to their category ‘*Cupressinoxylon* G’. The diagnosis of *Cupressinoxylon* Göppert (Göppert, 1850) has been emended by Dolezych (2005: 137).

Our wood shares the anatomical characteristics discussed by Dolezych (2005). The examination of the ‘*Cupressinoxylon* G’ category (Vaudois & Privé, 1971) has provided the following fossil species: *C. luckense* (Kostyniuk) Kräusel, *C. secretiferum* Greguss, *C*. sp.2 Grabowska, *C*. sp.1 Kostyniuk (Kostyniuk, 1967) and *C. matromnense* Grambast. The comparison of the latter species with our fossil wood has provided the following comparisons:

*C. luckense* (Kostyniuk) Kräusel (Kräusel, 1949) first described as *Cupressinoxylon* sp. in Kostyniuk (1938), has shorter uniseriate rays (up to 12–cells high), no indentures, and ray tracheids, only uniseriate bordered pits and absence of crassulae, and only smooth and slightly thickened tangential walls of the axial parenchyma. *C. secretiferum* Greguss has strange, big resin canals, up to 20 cells ray height (Greguss, 1967), and with *Cupressinoxylon* sp.2 Grabowska (Grabowska, 1957) share nodular axial parenchyma walls. *Cupressinoxylon* sp.2 Grabowska (Grabowska, 1957) has short rays (up to 10–cells high). The cross–field pits of *Cupressinoxylon* sp.1 Kostyniuk (1967) are larger than in the studied sample (6–10 μ in diameter). It has horizontal and tangential walls of smooth and sporadically pitted ray parenchyma and no indentures. Our wood is assigned to *Cupressinoxylon matromnense* Grambast, from the late Oligocene of the Paris Basin (Grambast, 1952), attributable to our fossil wood lacking callitroid and juniperoid thickenings, the presence of 1–4 (rarely 5) cross–field pits with “large apertures”, axial parenchyma with transversal walls that are smooth and pitted, bordered pits that are uni–bi–seriate, ray parenchyma walls that are smooth, the occurrence of indentures, and the average height of the rays is less than 20 cells.

If one takes into account Kräusel’s key of *Cupressinoxylon* species (Kräusel, 1949: 174) then the possible affinities are *Cupressinoxylon sabinianum* Schenk or *C. discoense* Walton. *Cupressinoxylon sabinianum* was initially identified by Beust (1884) as *Libocedrus sabiniana* Heer and then transferred to *Cupressinoxylon* by Schenk (*Schenk in Zittel, 1890*). The main differences of *Cupressinoxylon sabinianum* Schenk from our wood are the occasional occurrence of pitted horizontal walls in ray parenchyma and the short rays of up to 5 cells in height. *C. discoense* Walton (Walton, 1927) has an abrupt transition from early to latewood tracheids, and an absence of tangentially zonate axial parenchyma. The age of *C. discoense* is probably Cretaceous and it is almost identical to *Cupressinoxylon* sp.2 Zalewska (Zalewska, 1953), with the exception of up to six cross–field pits in the last species that resembles possibly *Widdringtonioxylon* Greguss (Vaudois & Privé, 1971; Philippe, Zijlstra & Barbacka, 1999).

Wood similar to our findings have been reported in the fossil record. Dupéron-Laudoueneix (1979) described a wood very similar to our sample: *Cupressinoxylon eocenicum* Dupéron–Laudoueneix. This wood is identical to ours except that it has mainly quadrangular tracheids and most importantly the transition between the early– and latewood is abrupt. *C. eschweilerense* van der Burgh (van der Burgh, 1978) has rounded tracheids but lacks indentures and the number of the cross–field pits unfortunately is not indicated. The last two characteristics are important for the assignment of our specimen to *Cupressus arizonica* as proposed by van der Burgh (1978). van der Burgh (1978, 1986) has indicated that *C. arizonica* wood is similar to both *Chamaecyparixylon polonicum* (Kräusel) Chudajberdyev, formerly *C. polonicum* Kräusel (which has rays >10 cells high; Vaudois & Privé, 1971), and *Cupressinoxylon* sp.2 Grabowska (which has rays <10 cells high; Vaudois & Privé, 1971).

*Cupressinoxylon xanthocyparioides* Dolezych (Dolezych & Schneider, 2006) also was similar to our wood specimen, differing in several characteristics such as the absence of indentures, smaller cross–field pit apertures, and cross–field pits present in one row however, it should be emphasized that this fossil took its name from its botanical affinities with *Xanthocyparis nootkatensis* (D. Don) Farjon & D.K. Harder, which has been renamed as *Chamaecyparis nootkatensis* (D. Don) Spach (or *Callitropsis nootkatensis* (D. Don) Florin ex D.P. Little, or *Cupressus nootkatensis* D. Don).

Both *Cupressinoxylon* sp.1 and *Cupressinoxylon* sp.2 have nodular transverse end walls of parenchyma and frequent or rare indentures, respectively, but they have small cross–field pit apertures (Vaudois & Privé, 1971).

Özgüven-Ertan (1977) described *Cupressinoxylon akdiki* Özgüven–Ertan from the Pliocene of western Turkey (Manisa–Soma locality), and the wood has differently developed traumatic axial resin canals, larger bordered pits (14–16 µm), and pits on radial walls of tracheids that are 1–3 seriate.

A *Cupressinoxylon* wood from the same locality was identified as *Cupressinoxylon pliocenica* Akkemik (Akkemik, 2020). *Cupressinoxylon pliocenica* differs from our specimen in having rather wide axial parenchyma cells in radial and cross sections, irregularly widened and very large ray cells in tangential section, and predominantly smooth end walls of axial parenchyma cells.

Based on the above discussion, we identified the fossil wood as *Cupressinoxylon matromnense* Grambast of the middle Miocene age from Gökçeada Island.

**Botanical Affinities.** The following anatomical characteristics of our fossil wood are important for ascertaining its botanical affinities: (a) cupressoid cross–field pits of (1–) 2–3 (–5); (b) for ray cells, the end walls of rays are generally smooth and horizontal walls may be smooth and dentate; and (c) for axial parenchyma, the end walls of our *Cupressinoxylon* sample are smooth, irregularly thickened, and nodular in radial section. The following anatomical character codes from Esteban et al. (2004) describing our *Cupressinoxylon matromnense* specimens (AT1, AT3, AT6, AT10, AT11, AT26, AT28; P2, P3, P5, P6, P7, P8; R1, R2, R4, R8, R16, R18, R24, R29, R37; RC1) all occur in *Cupressus* L. (*Cupressus* L. and *Hesperocyparis* Bartel & R. A. Price), *Xanthocyparis* L., and *Chamaecyparis* Spach (*Callitropsis* Oersted *sensu* Adams, Bartel & Price, 2009).

It appears that the co–occurrence of both smooth and nodular end walls of ray parenchyma can be regarded either as a special ontogenetic character related *e.g*., to juvenile woods (IAWA Committee, 2004); or as a character related to particular genera, *e.g*., *Glyptostrobus* (Visscher & Jagels, 2003), *Fitzroya* (Gasson, Baas & Wheeler, 2011), *Cryptomeria*, *Diselma*, *Fokienia*, and *Sequoiadendron* (Esteban et al., 2004); or even to genera of a particular region, *e.g*., North American *Cupressus* (Román-Jordán et al., 2016). Regarding the last–mentioned genus, the molecular phylogenetic analysis of *Cupressus* (*sensu* lato) by Adams, Bartel & Price (2009) has revealed a new genus for the North American *Cupressus* species: *Hesperocyparis* Bartel & R. A. Price.

Additional support regarding the distinction of the western *Cupressus* clade from the eastern clade has been provided by xylotomical studies. Román-Jordán et al. (2016) have found a link between the wood anatomical characteristics and the biogeographic distribution of Cupressaceae based on observations of specific characteristics, namely the arrangement of the axial parenchyma, transverse end walls of the axial parenchyma, presence or absence of ray tracheids, and typology of the end walls of the ray parenchyma cells and ray height. Based on their study, species with smooth, irregularly thickened, and nodular end walls of axial parenchyma are related to taxa in the North American region. In this region species have end walls of rays that are generally smooth and horizontal walls that are smooth and dentate, and up to 3 cupressoid pits per cross–field. By contrast, Eurasian species have only diffuse axial parenchyma with smooth end walls and only smooth end and horizontal walls of ray parenchyma.

The species from the North American clade, formerly assigned to *Cupressus* L., with ray tracheids are *Hesperocyparis arizonica* (Greene) Bartel, *H. bakeri* (Jeps.) Bartel, *H. lusitanica* (Mill.) Bartel, and *H. macrocarpa* (Hartw.) Bartel. There also is an interesting wood from the Eurasian region that has several similarities with the North American taxa, including the presence of ray tracheids in *C. duclouxiana* Hickel.

Following the anatomical features described in Román-Jordán et al. (2016), we arrived at the conclusion that our wood shares the majority of the characters including distinct growth ring boundaries; gradual transition from early– to latewood, axial parenchyma that is diffuse and marginal with smooth, irregularly thickened and nodular transverse end walls, ray tracheids, horizontal and end walls of ray parenchyma that are mostly smooth, presence of indentures, cupressoid cross–field pits, height of rays up to 15 cells, and uniseriate rays (with the extant *H. macrocarpa* (Hartw.) Bartel (formerly *C. macrocarpa* Hartw.)), found today in a very restricted native distribution in the Monterey Peninsula of coastal California (Farjon & Filer, 2013: 78). The only small differences are found in the existence of up to four (very rarely five) pits per cross–field, a characteristic found in *Xanthocyparis vietnamensis*, in our fossil wood specimen and that our wood has partial bi–seriate rays, a character implying an ecological dimension which is further analyzed and discussed below.

The special anatomical characteristics of our wood, and especially the axial and ray parenchyma walls can be used for its assignment to New World cypress trees (=*Hesperocyparis*) and in particular to *H. macrocarpa*, with only one different character. The only different character is the presence of up to four (or five) cupressoid cross–field pits which is more closely related to *Xanthocyparis vietnamensis*.

Several works on *Hesperocyparis* have been published recently (*e.g*., Adams, Bartel & Price, 2009; Terry, Bartel & Adams, 2012; Terry et al., 2016; Leslie et al., 2018; Zhu et al., 2018; Yang, Ran & Wang, 2012; Román-Jordán et al., 2016, 2017) that provide information about the capacity for dispersal of this genus over great distances during the past, in contrast with the extant species. Migration from Asia across Beringia is borne out, providing different ages for the possible divergence between *Callitropsis nootkatensis*–*Xanthocyparis vietnamensis* lineage and the New World cypresses (NWC): late Eocene according to Mao et al. (2010) and Yang, Ran & Wang (2012); middle Miocene *sensu* Leslie et al. (2012); and middle Eocene as supported in Terry et al. (2016).

Combining our microscopical observations with the literature we could state that our fossil wood material from the middle Miocene of western Turkey (eastern Mediterranean) conforms with the majority of the anatomical features of *Hesperocyparis macrocarpa* (and *Callitropsis nootkatensis*) combined with an anatomical characteristic of *Xanthocyparis vietnamensis*.

The occurence of a specimen from the basement of Marne river in Paris, *Cupressinoxylon matromnense* Grambast, is related to reworked material and has a late Oligocene (Chattian) age (28–23 Ma). Furthermore, *Cupressinoxylon eocenicum* Dupéron–Laudoueneix from the Eocene of Charente, France, was initially related to *Cupressus (Hesperocyparis) macrocarpa* by Dupéron-Laudoueneix (1979), although some characters, like the abrupt transition between the early and latewood tracheids may be substantially different from the anatomy of *Hesperocyparis macrocarpa* (Román-Jordán et al., 2016).

Taking into account the divergence age among *Hesperocyparis*–*Xanthocyparis vietnamensis–Callitropsis nootkatensis* in Cupressaceae clades (Mao et al., 2010; Leslie et al., 2012; Terry et al., 2016), and specific anatomical characters namely: (a) the occurrence of gradual transition between the early and latewood tracheids; (b) the diffuse and tangential/marginal axial parenchyma with smooth and pitted walls; (c) the occurrence of indentures; and (d) the existence of up to four pits per cross–field, we would like to support the assignment of the above-mentioned fossil wood species from Gökçeada, Paris, and Charente as stem lineage or as an extinct lineage of the *Hesperocyparis*– *Xanthocyparis vietnamensis–Callitropsis nootkatensis* clade (or HCX Clade *sensu* Terry et al., 2016), providing evidence for an ancestral lineage in central Europe and western Asia since the late Palaeogene.

Table S1 lists the possible botanical affinities of the species assigned to *Cupressinoxylon* E and *Cupressinoxylon* G *sensu* Vaudois & Privé (1971), and the *Hesperocyparis*– *Xanthocyparis vietnamensis–Callitropsis nootkatensis* clade with 1–4 cupressoid cross–field pits and axial and ray parenchyma walls related to the New World cypress trees should be further investigated with the goal of ascertaining their botanical affinities.

An issue that needs evaluation is the measurements of the cross–field pits. According to the IAWA Committee (2004: 54) codes 96–100, one of the microscopic features for conifers identification is the most common number of cross–field pits based on more than 25 counts. Alternatively, taking into account the study of Román-Jordán et al. (2017: 208–209), the number of pits is not included for those features considered to have diagnostic value. The authors of the present article would like to draw attention to this matter using the present case study as an example of the necessity of such an observation both in fossil and extant material: the most common occurrence of the cross–field pits in our case is two to four. This anatomical feature was among the specific characteristics which finally led us to understand that our wood specimen is a stem–lineage relative or an extinct lineage of the crown stem clade *Hesperocyparis*–*Xanthocyparis vietnamensis–Callitropsis nootkatensis* and not a member of the *Hesperocyparis* clade alone.

The present article also supports the work by Román-Jordán et al. (2016) regarding the differentiation of the *Cupressus* clades based on the wood anatomical characteristics. This is a perspective not discussed by Little (2006) who believed that the distinction between the New and Old World cypresses cannot be based on a single morphological feature.

**Plant–agromyzid associations**

Family Agromyzidae (Diptera) Fallén, 1823

Genus PROTOPHYTOBIA Süss, 1979

***Protophytobia* sp.**

**Figure 6:**
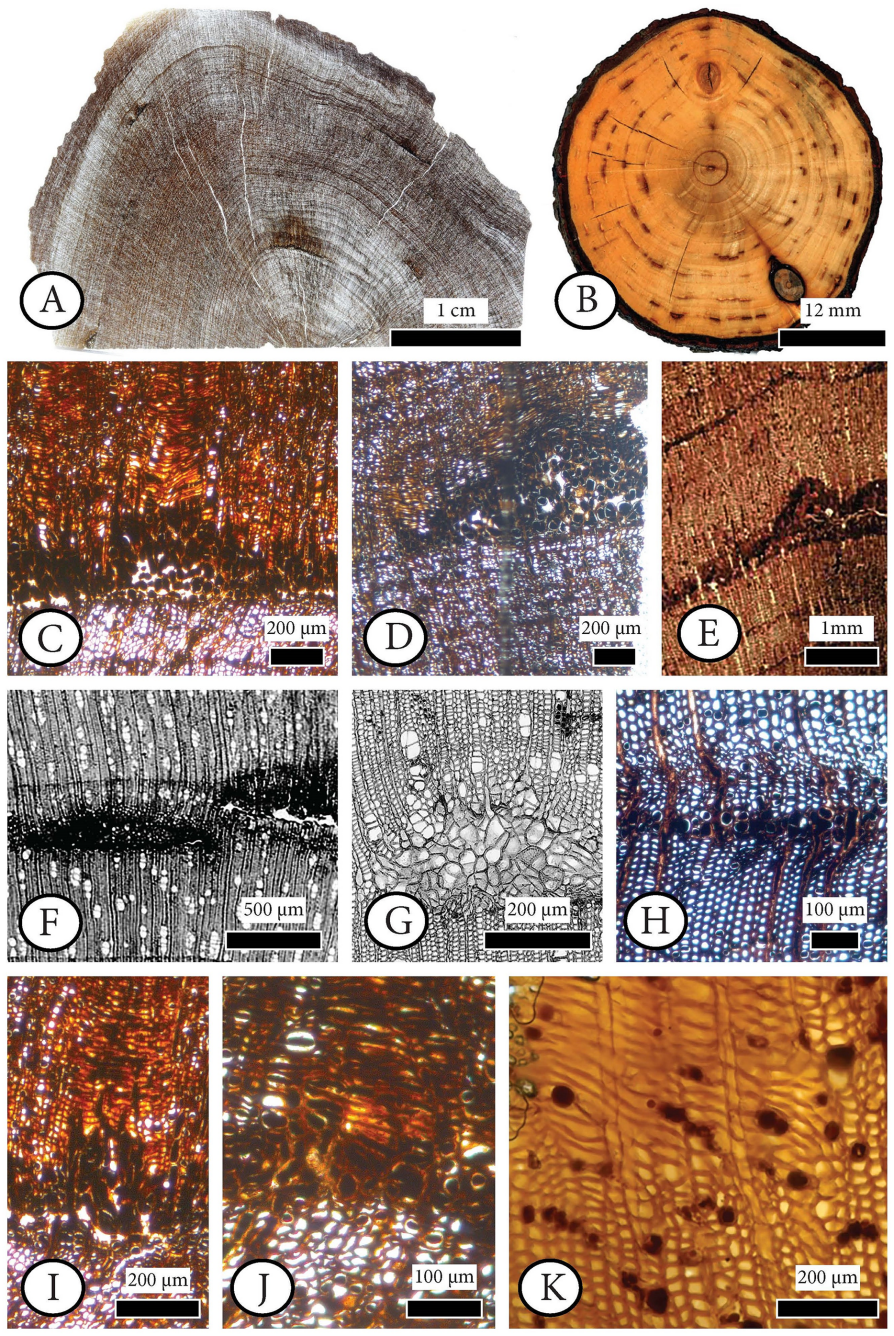
*Cupressinoxylon matromnense* Grambast from Gökçeada, Turkey (A, C, D, H, I, J) with *Protophytobia* Süss and adventitious shoots and fossil and extant material for comparison. (A) Growth ring boundaries and pith flecks or medullary spots observed in low magnification in transverse in our fossil wood. (B) *Phytobia cambii* (Hendel), larval pith flecks or medullary spots in a basal cross section of a young willow tree (*Salix* spec., Bielefeld, Germany, photo by M. von Tschirnhaus), from Černý et al. (2018: 36, Fig. 11). (C, D) Wounds in former cambium, reported as pith flecks including enlarged cells with dark content. (E) Damage types similar to ours, observed in *Circoporoxylon barnimense* Süss & Knöfler from Brandenburg, Germany, from Süss & Knöfler (2013: Taf.I, Fig. 2). (F, G) Traces of cambium damaging insects: pith flecks caused by insect feeding, from Schweingruber (2007: 214–216, Fig. 8.65, 8.67b). (H) Damage through the former cambium. (I, J) Elongated parenchymatic cells directly originating from a pith fleck (or cambium ‘pocket’ formation), being related with reaction processes: callus and adventitious shoots formation. (K) Elongated parenchymatic cells not directly originating from a pith fleck (*Juniperoxylon silesiacum* (Prill) Kräusel, from Limburg, the Netherlands. Material: Schönfeld’s fossil collection, no H70.2, SNSD). (A–K): transverse section.

**Figure 7:**
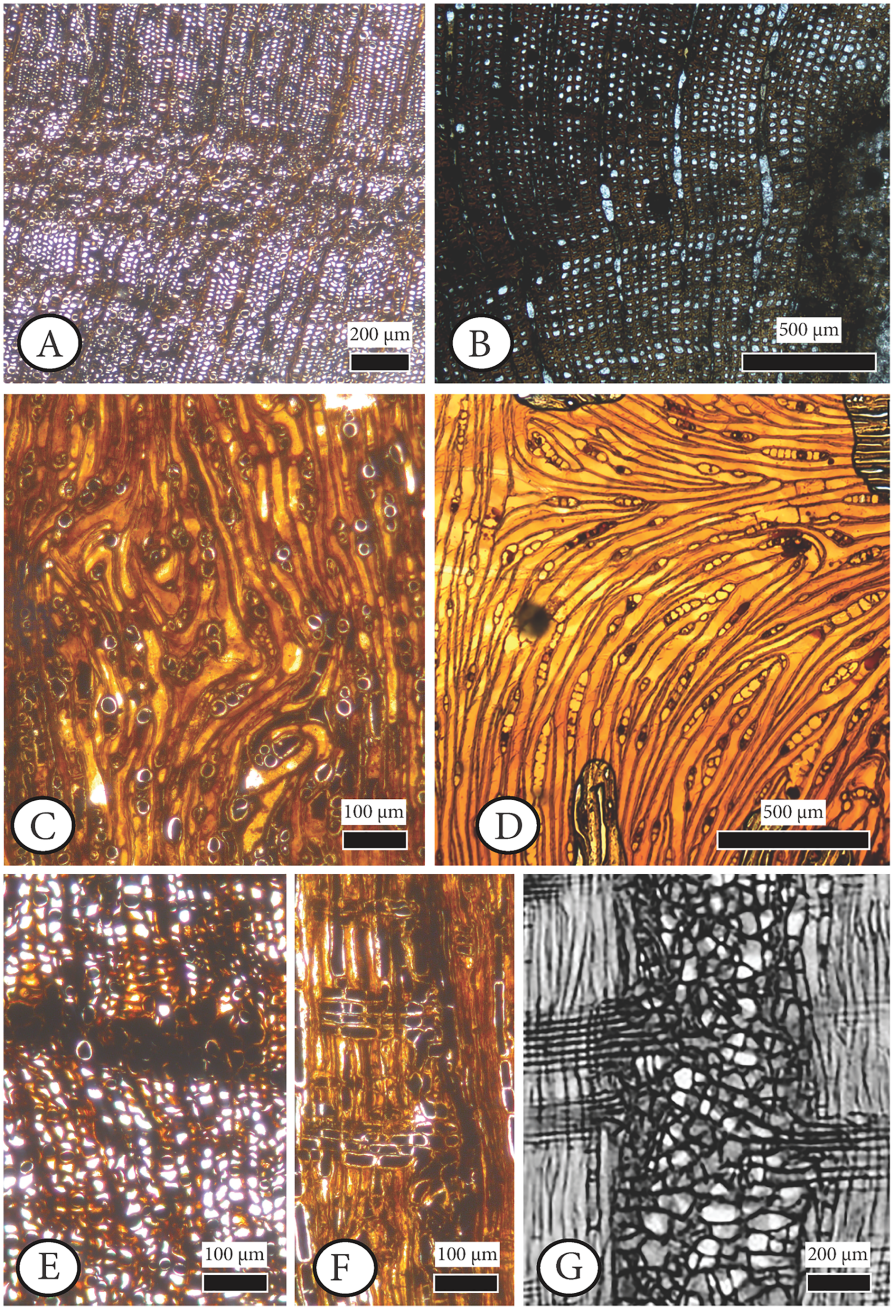
Anatomical abnormalities in *Cupressinoxylon matromnense* Grambast from Gökçeada, Turkey (A, C, E, F) and *Juniperoxylon silesiacum* (Prill) Kräusel from Limburg, the Netherlands. (A) Not continuous growth ring boundaries. (B) Discontinuous ring boundaries (*Juniperoxylon silesiacum* (Prill) Kräusel, Schönfeld’s fossil collection , no A10, SNSD). (C) Whirled zones, following a spiral girdle. (D) Whirled zones, following a spiral girdle (*Juniperoxylon silesiacum* (Prill) Kräusel, Schönfeld’s fossil collection, no A10, SNSD). (E) Wound in the former cambium as seen in transverse view. (F) The same wound (of Fig. E) as seen in radial view, probably assigned to *Protophytobia* Süss. (G) Traces of cambium-damaging insects: rays and cambium cells during callus production as a response to the damage, from Schweingruber (2007: 214–216, Fig. 8.66).

**Figures 6, 7, Table S2.**

**Material.** GOK17 (specimen and thee slides)

**Repository.** Istanbul University–Cerrahpaşa, Forestry Faculty, Department of Forest Botany,

**Locality.** East of Eşelek Village–Gökçeada, Turkey.

**Age.** middle Miocene.

**Host.** *Cupressinoxylon matromnense* Grambast, 1952

**Diagnosis.** Pith flecks/scars both inside and between the growth rings of *Cupressinoxylon matromnense* Grambast from Gökçeada, Turkey. The observed radial widths of the scars are 0.1–1 mm, and tangential diameters 0.5–3.3 mm. The dimensions of the hypertrophic cells with dark content are measured as follows: Radial diameter is 41.8 (17.1–69.0) µm, tangential diameter 34.9 (20.7–62.8) µm, and cell wall diameter is 6.1 (3.5–11.0) µm.

**Comparison with material from the fossil record.** Pith flecks of this size and position, both inside and between the growth rings (which used to be initially the former cambium) have been observed in the fossil wood record and were interpreted as feeding canals produced from the larvae of cambium miners belonging to fossil *Phytobia* Lioy (Agromyzidae, Diptera), including two fossil genera: *Palaeophytobia* Süss & Müller–Stoll (Süss & Müller–Stoll, 1975) for angiospermous hosts and *Protophytobia* Süss (Süss, 1979) for coniferous hosts.

The genus–host specification circumscribes the life habit of extant *Phytobia* Lioy that led to the identification of several species of this insect in the fossil wood record:

*Palaeophytobia platani* Süss & Müller–Stoll (Süss & Müller–Stoll, 1975) was identified in *Platanoxylon hungaricum* Süss & Müller–Stoll, *P. palibacsii* Süss & Müller–Stoll, and *P. sarmaticum* Süss & Müller–Stoll from the late Miocene of Hungary, *Palaeophytobia prunorum* Süss & Müller-Stoll (Süss & Müller-Stoll, 1980) was identified in *Pruminium gummosum* Platen emend Süss & Müller–Stoll from the Eocene of Yellowstone National Park, Wyoming (USA); and *Palaeophytobia* sp. was identified by Süss & Müller-Stoll (1980) in *Maloidoxylon castellanense* Grambast-Fessard (Grambast-Fessard, 1966) from the Pliocene of France.

*Protophytobia* Süss (Süss, 1979) has been identified for *Juniperoxylon silesiacum* (Prill) Kräusel (Kräusel & Schönfeld, 1924) from the Tertiary of Limburg, the Netherlands. Indications about its occurrence in *Circoporoxylon grandiporosum* Müller–Stoll & Schultze–Motel from the early Jurassic of France and *Circoporoxylon barnimense* Süss & Knöfler from the early Jurassic of Brandenburg, Germany can be found in Süss & Philippe (1993) and Süss & Knöfler (2013), respectively. As our fossil host wood is a conifer, therefore the ichnogenus observed in this coniferous host should be *Protophytobia* Süss. Although we provide measurements regarding the pith flecks and the hypertrophic cells dimensions (Table S2), we follow the widely accepted understanding discussed in Süss & Müller-Stoll (1980: 354–358) that the size of the marrow spots is dependent on the development of the herbivorous larvae and can range from submillimeter to several mm in transverse section. However, there is not an extensive occurrence of these structures observed in the radial plane. Secondly, there are brown-coloured hypertrophic cells seemingly filled with gummy contents produced by the larvae from dissolving the parenchymatic cells, consisting of lysigenic cells connected by a network of intercellular ducts. For coniferous fossil woods, as in the case of *Protophytobia cupressorum* in *Juniperoxylon*, the occurrence of abnormal tracheids inside the food channels occur as brownish pith flecks as reported by Süss (1979).

The genus–host identification of *Phytobia* could lead us to propose a new species for our identification of *Protophytobia*. The identified intraspecific competition in some *Phytobia* populations (Ylioja et al., 2002) could also support such a decision. Nevertheless, we would like not to create a new species of *Protophytobia* at present and would like to increase the information for this taxon when more clues on the evolution, phylogeny, and especially evidence for its occurrence on extant cupressaceous hosts, especially those from Australia and New Caledonia, becomes available.

**Cambium mining by agromyzid flies, and its structural-environmental reactions**

The cause and ecological/environmental significance of the tissue damage and associated abnormalities observed in our fossil are interpreted in detail, as follows.

The evidence of arthropod herbivory on different fossil plant organs (Scott, 1992) can be observed and quantified based on investigations of damage types on leaves (*e.g*., Müller, Wappler & Kunzmann, 2018), seeds (*e.g*., Barbosa dos Santos, Pinheiro & Iannuzzi, 2020) and wood and other indurated tissues (*e.g*., Xiao et al., 2022a, 2022b), providing a broad variety of plant–insect interactions. Different parts of the tree may serve as hosts for different pest categories (Wylie & Speight, 2012: 92, Fig. 5.1).

When it comes to wood, Labandeira (2005) uses the term “boring” or “borings” (Xiao et al., 2022a, 2022b) for the feeding groups related to endophytic phases of phytophagy in woods. Scott (1992: 209) has distinguished wounding from boring traces, relating wounding with damage to a living plant made by an arthropod during its ectophytic phase including a plant tissue reaction, while boring is related to an alive or dead host plant serving for feeding and shelter mainly for arthropods’ endophytic phase usually without a plant reaction. Scott (1992) reports the earliest fossil wounding in Devonian and the first wood boring in the Lower Carboniferous.

In the present study, we have evidence for arthropod herbivory in fossil wood made during its endophase (‘boring’ *sensu* Scott, 1992) followed by the wood’s reaction (‘wounding’ *sensu* Scott, 1992). We follow the compilation of Winkler et al. (2010: 936) of fossil agromyzids *Protophytobia* and *Palaeophytobia* as agromyzid feeding damage (‘ichnogenera’).

In *C. matromnense* from Gökçeada scars and anatomical abnormalities were observed. Interestingly, Grambast (1952: 334–335), in his description of *C. matromnense* from France refers to a generally well–preserved specimen, and he observed abnormal parenchymatic pockets that were dissimilar to resin canals in transverse section: “*des cordons de cellules de parenchyme, parfois bordés d’un cercle de cellules régulières; quelques–uns sont verticaux, de plus nombreux horizontaux, mais ils n’ont ni la structure ni la régularité de répartition des canaux sécréteur*”. Moreover, he reports the occurrence of mycelial parasitic infections when he was referring to partially bi–seriate abnormal rays. Unfortunately the above–mentioned abnormalities are not figured in his work, and we did not have the opportunity to study microscopically this specimen, but a potential relationship with the observed wound scars in Gökçeada wood could be examined in the future.

Timell (1986), in a 3–volume work, has specified the anatomical features which can be found in normal wood of several genera as well as in compression wood that express therefore ecological and environmental aspects rather than taxonomic features. Among the features which could be affected and therefore could show abnormalities are the ray and axial parenchyma with more in quantity, the higher number of rays that are partly biseriate, and with abnormal sizes of cells; the occurrence of rounded tracheids; and the presence of intercellular spaces and spirals. The above-mentioned anatomical features are given (in red color in Table S1) for clarifying rigorously qualified and quantified measurements when a tree or its wood undergoes biotic or abiotic stress (*e.g*., this work) and is assigned to a new species. *Cupressinoxylon secretiferum* Greguss (Table S1) is regarded by Timell (1986: vol. I, 602) as an example of compressed fossil wood. The wound scars and anatomical abnormalities observed on our fossil wood and their potential reasons are discussed as follows.

***Non-continuous growth rings*.** Our wood specimen may have structural similarities caused by low temperature compared to those found by Kłusek (2014) in her observations of *Cupressinoxylon polonicum* (=*Chamaecyparixylon polonicum* (Kräusel) Chudajberdyev, 1958 *sensu* Vaudois & Privé, 1971) from Hebdów (Poland), demonstrated as ‘frost rings’. The occurrence of frost rings in the wood from Poland was a result of an interval of frost, which supported ectothermic vertebrates (Böhme, 2003).

The examination of a frost interval ‘imprint’ in our wood anatomy leads us to study extensive discussions on the occurrence of frost rings in extant wood by Rhoads (1923) and Timell (1986: vol III, 15.2.5). Although there are similarities in the damage, such as the distortion of large dark parenchymatic cells in Rhoads (1923) there also are differences, for example, the damaged rays in the transverse section in Rhoads (1923) to our observations. Moreover, additional palaeontological evidence from Gökçeada Island is needed for supporting such a hypothesis.

Similar but not identical damage to our material is elongate, narrow scars covering almost the entire stem, interpreted as lightning damage as described in Luthardt, Merbitz & Rößler (2018).

Additionally, in Larson (1994: 572, Fig. 10.40; 573, Fig. 10.41; 578: 10.43) there are two examples of wounds or disturbances that are very similar to our findings.

The first is described again as a frost injury to the cambial region of *Larix gmelini* var. *japonica* in which the disturbed tissue arose primarily from the rays and parenchymatous cells.

The second one is a response to a moderate lightning injury in a *Picea abies* stem shown in the transverse section. In this region of the stem, the bark remained firm but gaps in the xylem were filled by proliferating ray and parenchymatous cells.

***Wounds in the former cambium*.** Arthropods such as termites, oribatid mites, and a variety of holometabolous insect larvae, particularly beetles, and macrofungi are the principle wood borers (McLoughlin, 2020; Schmidt, 2006; Scott, Stephenson & Chaloner, 1992). Larvae of beetles, especially the family Anobiidae (powderpost beetles) represent the most common insect wood–borings in the Cenozoic record (Rajchel & Uchman, 1998), and are found mainly in conifers as frass–filled tunnels variable in size and often of non–specific direction (although some anobiid borings show a preferential orientation along latewood/earlywood rings, C Labandeira, 2022, personal communications). Damage types from bacteria, fungi and arthropods have been reported from Lesbos fossil woods (Süss & Velitzelos, 2001).

The relatively small size of the scars in our wood sample along with their position exclusively in the former cambium (growth rings) and the absence of pelleted coprolites exclude beetles, mites, and termites from possible pests (McLoughlin, 2020; Labandeira, Phillips & Norton, 1997; Feng et al., 2015; Feng et al., 2019; Rozefelds & De Baar, 1991; Rajchel & Uchman, 1998; Zhiyan & Bole, 1989). Similar wounds related to cambium interpreted as ‘cambium mining’ are assigned to insect herbivory, reported as wood borings or medullary spots with ergastic deposits in Zavada & Mentis (1992: 9, Fig. 5B), or as pith flecks or tracheids with altered features (Süss, 1979), or parenchyma flecks (Rexrode & Baumgras, 1980).

Cambium mining is mainly related to a specific life habit (endophase) of specific families of holometabolous insect borers (Solomon, 1995: 621). Agromyzidae (Diptera: *Phytobia* Lioy) are regarded as cambium miners with a host–specialized mouthpart including two subapical teeth conjoined into a single structure (Labandeira, 2005: 223; Labandeira, 2019: 641) forming parenchyma or pith flecks during their endophase (Solomon, 1995: 621, Fig. 240.E; Winkler et al., 2010: 938, Fig. 1). Agromyzid larvae (*i.e*., maggots) are fluid feeding, as they have mouth hook mouthparts that puncture individual cells to imbibe their protoplasts (Labandeira, 2019).

***Enlarged (hypertrophic) cells with dark content*. **Enlarged (hypertrophic) cells with dark content at the end of the latewood and beginning of the earlywood zone which could be of six forms. First, are the sickle–shaped nodules from viral and bacterial infections of adventitious shoots and roots introduced in the tree (*e.g*., Galatis, Katsaros & Apostolakos, 1998: 610–611).

Second, are bacterial and fungal infections induced by Diptera that transport fungi to the side of the creation of a lateral root. In such a case the infections were the cause for the creation of the lateral roots. Morris et al. (2021) report that the occurrence of melanins associated with wood decay by fungi involving Basidiomycota and/or Ascomycota is a wood defense mechanism, or related to a fungus undergoing highly localized chemical stress such as from copper sulphate. These authors indicate the ideal temperature, pH, oxygen, and moisture conditions for such decay.

Third, hyperplasic parenchymatic cells are formed as a hypersensitive response to root disease (Blanchette & Biggs, 1992: 188).

Fourth, related to the upcoming death of the tree when xylomycetophagous hymenopterans such as sawflies feed on symbiotic fungi farmed in their galleries (Rajchel & Uchman, 1998). Deyrup (1975: 1–2) dealt with such a case, stating that the hymenopteran species found in extant *Hesperocyparis macrocarpa* were *Sirex areolatus*, *S. californicus*, *Xorides insularis*, and *Helcostizus albator*. The interpretation of the borings of Hymenoptera (Rajchel & Uchman, 1998) is not in agreement with the observed disturbances in our wood.

Fifth is callus cells (Dujesiefken, Stobbe & Kowol, 2001) caused by the growth of a fungus. A modern analog is canker disease introduced by the fungus *Seiridium cardinale* on cypresses and *Xanthocyparis* (formerly *Chamaecyparis*) interpreted by Spanos & Woodward (1997) and Swart (1973). These cankers cause axial parenchyma cells to be rapidly filled with secondary compounds, suggesting a rot or predator deterrence (Carlquist, 2018: 258).

Sixth, are pith flecks largely filled by secondary growth of modified (sclerotic) tracheids as a result of larval tunnels, similar to those made by Opostegidae (Lepidoptera) and Agromyzidae of the (Diptera) (Davis, 1989: 6). A more detailed observation of pith fleck shape indicates that they are analogous to extant *Phytobia* Lioy traces (Bonham & Barnett, 2001), as has been observed and analyzed in fossil coniferous woods by Süss (1979) and Süss (1980).

Süss (1979) had described the occurrence of *Protophytobia cupressorum* in *Juniperoxylon silesiacum* (Prill) Kräusel from Limburg in the Netherlands. This fossil wood, identified by Kräusel & Schönfeld (1924), was revised by Greguss (1970: 270) as *Palaeocallitroxylon limburgense* Greguss (1970: 270) due to the observations of similar patterns on extant *Callitris*, which finally Süss (1979) demonstrated were dipteran traces and not taxonomic artifacts. Philippe, Zijlstra & Barbacka (1999: 671) stated also that *Palaeocallitroxylon limburgense* Greguss should not be distinguished from *Juniperoxylon silesiacum* (Prill) Kräusel (Kräusel & Schönfeld, 1924), but rather is regarded as a synonym of it. Currently, *Juniperoxylon silesiacum* (Prill) Kräusel is considered as a synonym of *Juniperoxylon pachyderma* (Göppert) Kräusel (Kräusel, 1949: 177; Vaudois & Privé, 1971).

The first observations regarding the abnormal findings of *Juniperoxylon silesiacum* (Prill) Kräusel can be found in Kräusel & Schönfeld (1924). Original preparations (Schönfeld fossil collection, specimen numbers A4, A10, E11, H41, H44, H58, H65, H69, H70.1, H70.2, H74, R14.3 of Kräusel & Schönfeld (1924)) housed in SNSD have been microscopically restudied for the purposes of the current study. Among the material studied by Kräusel & Schönfeld (1924), specific identifications included wounded wood, which, Süss (1979) later restudied and identified as *Protophytobia cupressorum*. This material is not included in SNSD’s collection. However, the possible anatomical abnormalities caused by this pathogen were examined microscopically, the preparations which we had at our disposal and were initially reported by Kräusel & Schönfeld (1924) as having a ‘maserholz’ pattern.

Our anatomical study showed that the term ‘maserholz’ of Kräusel & Schönfeld (1924) described whirled zones, possibly related to callus or tumor tissue (Schönfeld fossil collection, specimen number H70.2, Fig. 7D) as well as elongated parenchymatic cells (Schönfeld fossil collection, specimen number H70.2, Fig. 6K). The latter mentioned cells are not directly originating from a cambium ‘pocket’ formation, as in the fossil wood from Gökçeada (Schönfeld fossil collection, specimen number H70.2, Fig. 6K–Gökçeada wood Figs. 6A, 6C, 6D, 6I, 6J). Sclerotic tracheids (or ‘pseudosclerotial masses’ *sensu* Morris et al., 2021) associated with cambium zones were observed in several preparations (*e.g*., Schönfeld fossil collection, n^o^ H70.1; H65; A4). Partly injured growth rings related to frost events as seen by Kłusek (2014): *Cupressinoxylon polonicum* (=*Chamaecyparixylon polonicum* (Kräusel) Chudajberdyev, 1958 *sensu* Vaudois & Privé, 1971) were also observed (Schönfeld fossil collection, specimen A4) as well as discontinuous ring boundaries (Schönfeld fossil collection, specimen A10, Fig. 7B). Pith flecks related to *Phytobia* were not observed in this material.

Similar structures interpreted as ‘seemingly pith–flecks from wound wood’ (‘Markflecken–ähnliche Wundgewebebildungen’) were observed by Süss & Philippe (1993: Abb.3) in *Circoporoxylon grandiporosum* Müller–Stoll & Schultze–Motel from the early Jurassic of France, and by Süss & Knöfler (2013: taf. I, Fig. 2) in *Circoporoxylon barnimense* Süss & Knöfler from Brandenburg, Germany, probably of early Jurassic age. The latter authors stated that the occurrence of these wound types, especially restricted only to the inner growth rings, could potentially be related to *Protophytobia* (Diptera, Agromyzidae) and its herbivorous larvae, and not to other abiotic and biotic factors.

Based on our observations the damage types occurring in *C. barnimense* (Fig. 6E) that are very similar to the ones found in the wood from Gökçeada (Figs 6C and 6D), are related to *Protophytobia*, although the shape of the wounds is more triangular both in *Gökçeada* wood and in *Circoporoxylon barnimense* Süss & Knöfler compared to the more rectangular ones assigned to *Protophytobia* (and *Palaeophytobia*). Similar patterns of triangular pith flecks assigned to *Phytobia* Lioy are also seen in the woods examined by Kumada (1984).

The occurrence of marrow spots of *Protophytobia* in *Juniperoxylon silesiacum* is not restricted only to the cambium, as in *C. barnimense*, but also observed in the transverse section in the middle part of the growth zone up to the last latewood and into the earlywood of the following growth zone (Süss, 1979). These marrow spots are represented both in the fossil and extant *Phytobia’s* fluid–feeding habit (De Sousa & Couri, 2017; Geissert, Nözzold & Süss, 1981; Süss, 1979; Süss, 1980; Winkler et al., 2010: Fig. 1–1) and is also observed in *Gökçeada* wood, with scars fully measured as seen in *‘Pith flecks diagnose’* paragraph, and in Table S2 both inside and between the growth rings. More specifically, although it was believed that *Phytobia* mining larvae fed directly on cambium, it has been found that the larvae feed on young, undifferentiated cells right after their detachment from the cambium (Solomon, 1995; De Sousa & Couri, 2017), a feature regarded as plesiomorphic, resulting in a long history of this genus (Spencer, 1990).

Moreover, Süss (1979) reports that the irregularly arranged tracheids, occasionally filled with brown substances, were involved in wound tissue formation, a characteristic observed also in our wood. An investigation on the extant record of *Phytobia*, reveals that triangular scars have been reported in *Betula* woods affected by *Phytobia* (Bonham & Barnett, 2001) but no other cambium miners with such a specific gallery construction, except *Protophytobia* Süss, have been reported in coniferous hosts from the Cenozoic (Süss, 1979; Süss, 1980). Damage of wood xylem assigned to *Opostega* Zeller (in Winkler et al., 2010: Fig. 1–2, Lepidoptera: Opostegidae) does create similar patterns with *Phytobia* Lioy, with the only difference that the opostegid mines are less elongate than *Phytobia* mines in cross-section.

***Pericyclic parenchymatic cells and creation of lateral or adventitious roots*.** The observed pericyclic parenchymatic cells occurring at the top of the pith flecks could recall the following three modes of producing lateral or adventitious roots.

**Figure 8:**
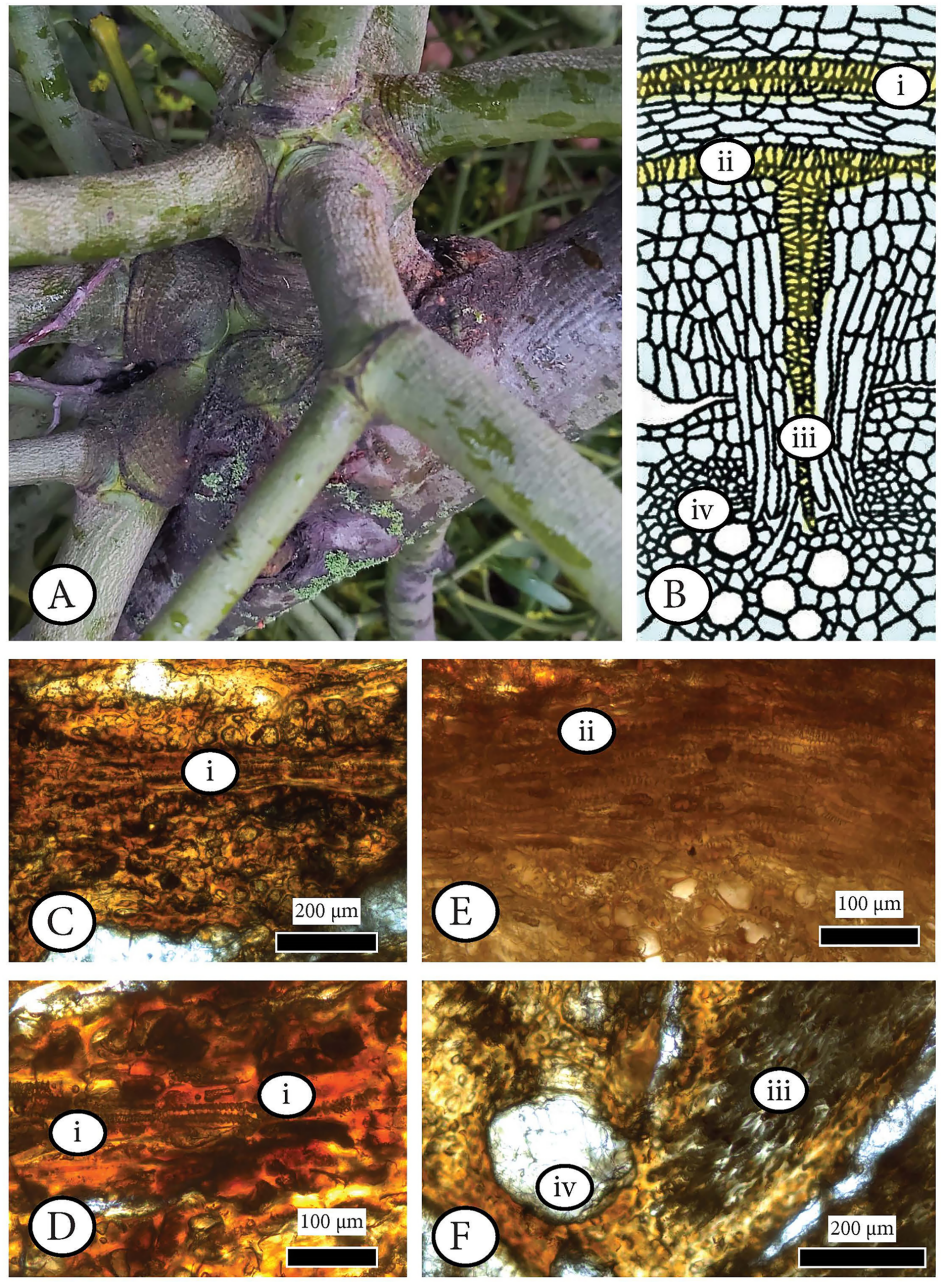
Examining the infestation of a mistletoe (*Viscum album*) in a host (*Pyrus malus*) as a possible analogue of the observed anatomical patterns in *Cupressinoxylon matromnense* Grambast from Gökçeada, Turkey. (A) Extant hemiparasitic *Viscum* on an angiosperm host (macrophoto). (B) Haustorium of *Cuscuta* parasite inside a stem of a host plant (from Pazourek & Votrubová, 1997: 53, modified). (C–F) Anatomical observation of the extant hemiparasitic *Viscum album* in *Pyrus malus* (Material: Schönfeld recent coll., no 29, SNSD). The anatomical study of this material is directly related to Fig. 6B denoting the process of infestation of a mistletoe inside a host’s xylem. The microscopic photos (C–F) correspond to specific anatomical positions (i–iv) during the infestation as shown in Fig. 6B. Figure 6D is an enlargement of Fig. 6C. (C–F): transverse section.

First is the infestation of a hemi–parasitic or parasitic plant such as mistletoe (Fig. 8A) as described in Teixeira‐Costa (2021), Teixeira-Costa, Davis & Ceccantini (2021), Schweingruber (2007), Pazourek & Votrubová, (1997: 53; Fig. 8B), Srivastava & Esau (1961a, 1961b), and Peirce (1893, 1905) with our fossil being the host, as in the example of *Cuscuta*, *Arceuthobium or Viscum* (Fig. 8B), with effects in coniferous host features, especially rays. An anatomical comparison with our wood does not support a possible co–occurrence of *Arceuthobium* as described in Srivastava & Esau (1961a) and Srivastava & Esau (1961b) because of differences in rays and potential sinkers (mistletoe stems that grow on outer xylem surface of the host’s branch or trunk).

A microscopic study of the anatomy of the hemiparasitic *Viscum* observed in the host (*Pyrus malus*) shows that the structure of haustoria (parasitic roots) (Schönfeld recent collection, specimen 29, Figs. 8C–8F) differs from our fossil wood’s parenchymatous cells (Figs. 6C, 6D, 6I, 6J).

Second, are cells of active cambial zones (as seen in Larson, 1994: 592, Fig. 11.5B), in ‘mother’ and ‘daughter’ xylem cells as discussed in Wilczek‐Ponce, Włoch & Iqbal (2021), or suberized parenchyma cells (Pearce, 1996: 210, Fig. 6 (BZ); Yamada, 2001: 131, Fig. 3a).

Third, are adventitious roots from plants damaged by volcanic ash falls as a strategy of revitalization such as rooting in the tuff bed. They have been reported both in the fossil record (Opluštil et al., 2007) and in recent times after volcanic eruptions (Smathers & Mueller-Dombois, 1972), for example, in extant *Chamaecyparis nootkatensis* after 2–4 months directly after the Mount St. Helens eruption of 18 May 1980, and in *Abies amabilis, A. procera, A. lasiocarpa*, *Thuja plicata*, *Tsuga heterophylla*, *T. mertensiana*, and *Pinus monticola* a year after the above–mentioned eruption (Zobel & Antos, 1982). Del Tredici (2001) uses the term “opportunistic sprouting” for expressing the importance of the adventitious shoots for regeneration (and vegetative propagation) when specific environmental conditions are met, also for the evolutionary traits of significance for the survival of the tree. This mechanism provides evidence on the relationship of regeneration and vegetative propagation present on fire–(or non)–adaptive genera according to the hemisphere, discussed also in Bond & Midgley (2003: 108); non-fire-adapted sprouting forests, possibly related to a more ancient sprouting mechanism: in the northern hemisphere only Taxodiaceae and Taxaceae show a collar root sprouting behavior and in the southern hemisphere *Arthrotaxis* D. Don and several genera of Podocarpaceae, such as *Lagarostrobos* C. J. Quinn and *Phyllocladus* L. C. Richard ex Mirbel, which do not burn, and fire-adapted sprouting forests: species of *Pinus* and in the sourthern hemisphere members of Cupressaceae and Podocarpaceae of fire-prone ecosystems. Consequently, their observations of the fossil record could represent much more than a fingerprint scenario, suggesting a possible connection between the occurrence of sprouting and a regeneration mechanism created in specific circumstances. Nunes et al. (2019) who discuss the finding of such a mechanism in Cupressaceous wood from the lower Cretaceous of Central Patagonia point out the absence of these findings in the fossil record. Additionally, Schweingruber (2007: 205) reports the formation of adventitious roots, observed along with narrow tree rings in trees that are about to die, after the burial of stems by volcanic ash.

The creation of adventitious roots (rhizogenesis) differs from the formation of lateral roots because the latter can be accomplished also from aerial tissues (*e.g*., stems) and it is not only an adaptive reaction to stress conditions but also “a key limiting component of vegetative propagation” (Bellini, Pacurar & Perrone, 2014). This last observation, in combination with Rexrode & Baumgras (1980)’ study that the quantity of the pith flecks is related to the height of the tree, could provide a possible explanation for the branch origin of our findings and therefore the occurrence of adventitious rather than lateral roots.

***Callus formation associated with cambium damage and formation of adventitious roots and shoots*.** Callus or tumor formation is clearly seen in longitudinal sections (Fig. 7C) of our wood where whirled (or swirled) zones are observed to follow a spiral girdle (as discussed also in Schweingruber, 2007 and in Larson, 1994: 522, Fig. 10.13B).

A relationship between the callus structure and *Phytobia* pathogeny can be found in Spencer (1990: 15) “*Coleopterous and lepidopterous larvae also feed in the cambium of living trees but a distinctive feature of Phytobia feeding is the development of callus cells in the feeding channel which becomes completely occluded, and with experience, such feeding tracks can with confidence be ascribed to Agromyzidae. In a section of an infested stem larval feeding of Agromyzidae is detectable by brownish marks which are now widely referred to as pith flecks.”*

Fink (1982) correlated the creation of adventitious root primordia with the presence of abnormally broad xylem rays both in angiosperms and conifers and also observed in our wood, (Figs. 4D and 4E).

Evert & Eichhorn (2006: 183) associates the development of adventitious roots, together with hypertrophied lenticles, with a significant abiotic stress factor such as rapid flooding. Hahn, Hartley & Rhoads (1920) examined the relationship between adventitious roots and hypertrophied lenticles with flooded conditions in conifers. Their final remarks were first, the existence of hypertrophied lenticles only on the basal portions or submerged parts of stems when abnormally wet situations occur; and second, the presence of hypertrophied lenticles when extreme humid situations occur occasionally on parts of the stems above the soil surface.

Consequently, the observed anatomical wood disturbances and reactions reflect potential biotic attacks (arthropods, fungi) and abiotic distortions (volcanism, light stress, rapid flooding) that the wood underwent millions of years ago. Schweingruber (2007: 220; Fig. 8.78a) discusses the interconnection of fungi with injuries and adventitious roots, stating that fungal spores “*enter the live tissue through wounds in the bark that were caused by tension or injuries. An attack affects the entire tree for several years. Below the necrotic spot, and on the stem basis, often adventitious shoots form. During the infection, abnormal structures form that would indicate problems with the water regime. Adventitious shoots are anyway the wood’s reaction to a severe damage/infection/stress*.” Our fossil wood material was diseased as is clearly seen by the overgrown dead parts of the cambium and the parenchyma cells in the water-conducting xylem that excretes dark, phenolic substances parallel to the tree rings (Schweingruber, 2007: 220, Fig. 8.77a,b).

Our geological data consisting of the wood entombed in volcanic ash susceptible to light stress and high temperatures was a result of volcanism, occurring as lahar flows along with the observed anatomical details of discontinuous growth rings, pith flecks filled with enlarged cells containing dark substances, parenchymatous cells of adventitious roots, and callus wood, have provided a glimpse into the past. Further work on the fossil woods from this locality could provide additional evidence in this respect and allow a palaeoenvironmental and palaeoecological reconstruction of the area.

## Discussion

Detailed studies on primary data can provide new evidence. Re-evaluating the coniferous wood findings from the Aegean for the purposes of our material identification, we realized nomenclature inconsistencies regarding a family name (Protopinaceae) and a fossil generic name (*Pinoxylon*) both used during the past decades (Süss & Velitzelos, 1993, 2010) for the identification/description of Greek and Turkish material. Bamford, Philippe & Thévenard (2016: 25), clearly explained that Kräusel (1917) proposed the term Protopinaceae during his postdoctoral studies for a fossil family “*based only on one single anatomical feature: a mixed type of pitting on tracheid radial walls*”, which had little phylogenetic relevance according to Bailey (1933). Two or intermediate types of radial pitting can co‒exist in the same specimen as proved also by the work of Philippe (1992). Bamford, Philippe & Thévenard (2016: 25, 28) concluded that *“the recent fossil wood anatomical (palaeoxylogical) papers using the term Protopinaceae for systematic purposes, e.g.*, Süss & Velitzelos (1993, 2010), *without a detailed justification, are misleading”*, explaining at the same time the Protopinaceae concept is invalidly published according to ICBN guidelines. This ICBN change clarifies the issue that Protopinaceae should never be treated as a separate systematic unit. For these reasons we suggest the cessation of use of Protopinaceae for the plant fossils from Greece and Turkey, and to employ the term Pinaceae in the case of *Lesbosoxylon* Süss & Velitzelos, and an uncertain position for *Chimairoidoxylon* Süss & Velitzelos. Moreover, the generic name *Pinoxylon* F. H. Knowlton in L. F. Ward 1900 is unstable while *Pinuxylon* W. Gothan 1905 is valid; therefore, we revise here the following species: *Pinuxylon parenchymatosum* (Süss & Velitzelos) Mantzouka & Akkemik (Table 1).

The special anatomical characters of this new *Cupressus* type of petrified wood, named here as *Cupressinoxylon matromnense* Grambast and especially the axial and ray parenchyma walls, could support its assignment to the New World Cypress trees of *Hesperocyparis*. The present petrified wood from the middle Miocene of the eastern Mediterranean matches the majority of the anatomical features of *Hesperocyparis* combined with the anatomical characteristics of *Xanthocyparis* (formerly *Chamaecyparis*) and thus could support the migration route of the genus *Hesperocyparis* while retaining its relationship with *Xanthocyparis*.

Comparison with the original material of *Juniperoxylon silesiacum*, the host of *Protophytobia cupressorum* Süss, has shown that most probably this taxon of Agromyzidae (*Protophytobia* Süss) was responsible for some of the observed damage types in Gökçeada wood. However, the possibility of another/new extinct species of *Protophytobia* remains open. Moreover, Süss (1979), Süss (1980), Süss & Müller-Stoll (1980), Spencer (1990), and Süss & Knöfler (2013) indicate that agromyzid cambium miners are genus host–specific; therefore, there is a possibility of the occurrence of another (extinct?) species of an agromyzid cambium miner more related to the *Hesperocyparis*–*Xanthocyparis–Callitropsis* clade and not to *Callitris*. Although genus–level host specificity is a widely accepted feature of the extant *Phytobia*, Nyman, Ylioja & Roininen (2002) suggest that co–speciation and strict parallel cladogenesis between the flies and their hosts cannot explain the evolution of these host–plant associations.

What is important here is to underline the fact that cambium mining evidence in fossil coniferous woods is currently restricted to the *Protophytobia* host: *Juniperoxylon silesiacum*. The observation of similar anatomical patterns in the early Jurassic *Circoporoxylon grandiporosum* Müller–Stoll & Schultze–Motel (in Süss & Philippe, 1993) from France and *Circoporoxylon barnimense* Süss & Knöfler (2013) from Brandenburg, Germany reveals new scientific question: If the findings from France and Germany are of Jurassic age, then they should not serve as hosts for an agromyzid such as *Protophytobia*, but a connection with Coleoptera, Hymenoptera, or Lepidoptera should be investigated, as well as a consistency with Herbivore Expansion Phase 3 *sensu* Labandeira (2006: 219–221). The Agromyzidae are a member of the broader Cyclorrhapha clade of Diptera that had its origin in the early Paleocene or the K–Pg (Cretaceous–Paleogene) boundary, based on a relatively rich fossil record (Labandeira, 2005, 2006; Winkler et al., 2010). Research on the stratigraphic situation and consequently the exact age of these *Circoporoxylon* species is crucial in order to illuminate a possible connection between *Protophytobia* and fossil Podocarpaceae (with *Circoporoxylon* from France and Germany as the host; Süss & Philippe, 1993; Süss & Knöfler, 2013), which could be true only in case of a younger age of the plant fossils. Until then, we support the host–specificity of *Protophytobia* on fossil Cupressaceae during Tertiary on the host: *Juniperoxylon* from the Netherlands, related to *Callitris*, in Süss, 1979, and an extinct cupressaceous wood from the *Hesperocyparis–Xanthocyparis–Callitropsis* clade from Gökçeada, Eastern Mediterranean, Turkey.

If one takes into account the taxonomic position of these fossils in combination with the defense mechanisms of extant coniferous families to insect herbivory such as the production of calcium oxalate (Hudgins, Krekling & Franceschi, 2003: 688, Fig. 7) and stone and polyphenolic parenchyma cells (Krokene, 2016: 1644, Fig. 1), then a possible cause of the absence of pith flecks in fossil coniferous woods record could be detected. Nevertheless, a more parsimonious and phylogenetically accurate reason is that Agromyzidae were not present in the Mesozoic (C Labandeira, 2022, personal communications). Alternatively, the coniferous hosts of both the fossil and extant *Phytobia*, namely Podocarpaceae and *Callitris* (N.B. *Actinostrobus* should be also investigated, see photos in Heady & Evans, 2005) currently inhabit the southern hemisphere. Consequently, their fossil occurrences, with the distribution of *Protophytobia* in such a different geographical context in contrast with extant findings–such as Gökçeada 40.1940° N and Limburg with 50.9213° N–could raise several questions regarding the evolutionary biology, differentiation of habitats, taxonomic preferences and/or the extinction of such biomes.

These observations could provide additional information on the Herbivore Expansion Phase 4 *sensu* Labandeira (2006: 421–423), regarding the host plants, their herbivores (*e.g*., Diptera), and the functional feeding group membership of the herbivores (borings). Given, this, the initial colonization of agromyzids could have been on angiosperms or on conifers, in which case there is the possibility that *Protophytobia* on conifers could be a secondary colonization event (C Labandeira, 2022, personal communications). Additionally, our findings and observations, which are the first ones in this region, could contribute to a chapter on the historical pattern of borings for insect damage types (Labandeira et al., 2007).

Concerning terminology, the observed pith flecks could be regarded as trace fossils but the plant reaction tissues such as the adventitious roots can not (Bertling et al., 2006). Further association of damage types observed in fossil wood and in leaves from the same assemblages would benefit from the knowledge of the mining insect trace fossil record (Bayless et al., 2021; Scheffer, Winkler & Wiegmann, 2007; Winkler, Mitter & Scheffer, 2009; Winkler et al., 2010), possibly providing more evidence regarding the holometabolous insect life cycle, particularly endophytic taxa in which wood serves as an enclosed medium for the egg, larval and pupal stages that provides for food and shelter *vs* ectophytic taxa, in which the adults live on leaf surfaces (Whitehill & Bohlmann, 2019). For Agromyzidae this was a pattern that was present from 66–60 million years ago (the earlier Paleocene) in a relationship with their hosts. The earliest evidence of holometabolous insects in fossil conifer woods were investigations on the evolution of ecological interactions of insects in wood involving a coniferous specimen from the middle Permian of Russia (Naugolynkh & Ponomarenko, 2010) and from the latest Permian of China (Feng et al., 2017), a miniature food web in which a polyphagan beetle is associated with a complex tunnel system of entry holes, mating gallery, larval tunnels, exit holes amid wood borers, fungi, fungivores, and predators. A similar type of beetle borings, without the associated food web, was described from the early Permian of Germany (Feng et al., 2019).

In this work, we discussed our observations regarding the fossil wood anatomical characters, their taxonomic evaluation, different damage types, their possible causes, and the wood’s reaction; thereby providing a window into these conifer–dipteran associations. Such exploration of the past is based on wood identification, systematics of wood anatomy, ecological aspects of wood anatomy *sensu* Carlquist (2001) or wood anatomical trait–based ecology *sensu* Beeckman (2016), and the associational approach *sensu* Labandeira (2005) for estimating the plant–insect–environment interactions.

Comparative wood anatomy has a wide range of subjects from wood to natural selection (Carlquist, 2001; Olson, 2012, 2020). According to Olson (2005: 507, 508) and Olson (2014: 8) *“comparative wood anatomy consists of two main efforts: wood identification (assigning names to samples) and evolutionary studies. Evolutionary studies can be divided into two main areas: wood anatomy (systematics) and ecological wood anatomy (identification of structure–function relationships)*.”

During our study we have examined our specimen in terms of wood identification and evolutionary studies, providing the systematics and ecological context based on wood anatomy from a detailed study of its special structure and function (*e.g*., reaction patterns). We also have discussed possible functions and reactions of wood facing specific predatory threats that are not related to ecological wood anatomy *sensu* Olson (2005).

Predation deterrence is not related to major trends in xylem evolution according to Carlquist (2001: 344); rather, our data involves a genus–host specific insect occurring since the early Paleocene or possibly K–Pg (Cretaceous–Paleogene) boundary, based on a relatively rich fossil record (Labandeira, 2005, 2006), providing a glimpse into the symbiotic past.

*Cupressinoxylon matromnense* from Gökçeada with *Protophytobia* sp. exhibits scars and other anatomical abnormalities, all of which were extensively analyzed with final remarks on the occurrence of the agromyzid larvae (*Protophytobia*) possibly with fungi (related to wounds in the former cambium with pith flecks, enlarged (hypertrophic) cells with dark content at the end of the latewood and beginning of earlywood zone), reaction tissues with the creation of adventitious shoots for regeneration (certified by the observed pericyclic parenchymatic cells occurring at the top of the pith flecks) related to high stress caused by biotic (insect invasion) and/or abiotic (volcanism and entombment and/or rapid flooding reflecting microclimatic conditions or regional climatic patterns related to MCO) factors.

Recent studies have proven that the relationship between wood anatomy and climate change is bidirectional (Anderegg & Meinzer, 2015; Wiemann et al., 1998). The more we focus on the understanding of the mechanisms through which climate affects xylem characteristics and plant hydraulics and the trees’ responses following this process as recorded in the Cenozoic the more knowledge and time we gain for predicting and protecting our planet’s future.

Moreover, the drastic climate changes affect the evolution of plant-animal interactions. The co-occurrences with remnants of fossil animals can provide significant evidence on the palaeoenvironment and palaeoclimate of the region as well as on the terrestrial food webs.

A correlation between mammal hypsodonty and grass-dominated habitats expansion is addressed in several works leading to a correlation with aridity, drying, cooling and increased seasonality globally (Saarinen, Mantzouka & Sakala, 2020) and can be applied in detail in a next study of ours: The finding of *Prodeinotherium bavaricum* von Meyer lower jaw teeth in Lesbos western peninsula (Gavathas), supported our knowledge for the early-middle Miocene of the eastern Mediterranean with data related to its biogeographic distribution, the age of its arrival and consequently dispersion from Africa to Eurasia (late MN3), an early connection between those two continents before the Tethyan Seaway’s closure through the “*Gomphotherium* land bridge” (Koufos, Zouros & Mourouzidou, 2003). The bilophont-brachydont teeth of this primitive form of the species are typical of forest and woodland dwellers.

Another hint of the subtropical palaeoenvironment of Lesbos island during the late early–early middle Miocene (MN3–MN4) is supported by the fossil micromammals’ teeth (*e.g*., *Eumyarion* Thaler, *Glirulus (Glirulus) diremptus* Mayr), the gastropod and ectothermic palaeocommunities, which, suggest the existence of a palaeolake inside a forest habitat (Mantzouka, 2009; Vasileiadou & Zouros, 2012; Vasileiadou et al., 2017).

Palaeontological findings from both Lemnos and Gökçeada may shed more light in this direction.

The combined evidence of terrestrial units with palaeobotanical species of the same crucial age, taphonomic history and preservation status belonging to the same geographic area are evaluated in this work as pieces of the same puzzle: a place of significance for the history of earth and the evolution of terrestrial life, namely North Aegean (Eastern Mediterranean) MCO hotspot.

Xylem anatomical studies provide useful information about the trees’ responses and adaptation strategies (structural and functional) as reactions to climatic changes in temperature and CO2 concentrations (Pandey, 2021). On the other hand, climate change affects both plants and animals and their interaction. The application of recent studies on extant material in the fossil wood record is beneficial for understanding the pre- and post- environmental conditions from the most valuable natural archives.

In this direction, the recognition of palaeo- and metacommunities was one of our next goals. Watkins, Berry & Boucot (1973) distinguished the local fossil biofacies from the fossil communities with taxa with related evolutionary lineages in space and time, known as palaeocommunities.

The metacommunities theory uses the four archetypes including species sorting, neutral, patch dynamics, and mass effects as the main categories of each community’s composition variety classification (Leibold et al., 2004) taking into account the internal structure of metacommunity ecology where environment, space, and co-distribution are dissected factors possibly related to species’ features and sites for examining the baseline colonization and extinction probability of species’ interaction (Leibold, Rudolph & Blanchet, 2020).

Global and regional climatic changes influence on metacommunities includes the macroevolutionary patterns, namely: local biotic and abiotic interactions and regional speciation, extinction, emigration, and immigration. Blanco et al. (2018) presented such a case study on highly distributed, diversified, and climatic sensitive Miocene rodent communities. The former work revealed two main affective factors of the correlation between metacommunities and long-term changes in the environmental and species characteristics. García-Girón et al. (2021) have applied the metacommunities concept for investigating the Mesozoic macroecological patterns of the dinosaur record of North America.

The latter two examples could be followed for the application of metacommunities dynamics (examination of possible patterns of compositional diversity and community–environment relationships) in fossil wood assemblages/sites.

## Conclusion

With this study, one more species of cypress, new for the palaeovegetation of Turkey, was added to the Miocene woody flora of Gökçeada Island. Its migration route to the Eastern Mediterranean area has been elucidated and the age of the fossil site has been assigned to the middle Miocene. The latter discovery led to the establishment of an eastern Mediterranean MCO hotspot including Lesbos, Lemnos and Gökçeada Islands and is not restricted to the area of the Petrified Forest of Lesbos Island. The cessation of Protopinaceae and the use of Pinaceae have been proposed, changing the nomenclature status of *Lesbosoxylon* Süss & Velitzelos and *Chimairoidoxylon* Süss & Velitzelos; the revision of *Pinuxylon parenchymatosum* (Süss & Velitzelos) Mantzouka & Akkemik has also been suggested.

The responsible culprit for the pith-fleck damage in the cambia of the identified wood was determined as *Protophytobia* sp. (Diptera: Agromyzidae). The identification of the new *Protophytobia* and the geochronologic timing and evolution of cambium borer taxa in Agromyzidae, and the primary or secondary colonization of certain conifer taxa were also detected. Known chemical and physical defense mechanisms of conifers related to the presence of agromyzid borers and other arthropod wood-boring lineages on conifers in the fossil record and across space were also discussed, with respect to the studied fossil wood. Finally, the role that conifer wood identification plays in the detection of the regional climate during the Neogene of the Eastern Mediterranean the region was also analyzed.

## Supplemental Information

10.7717/peerj.14212/supp-1 Supplemental Information 1 Comparison with the fossil *Cupressinoxylon* species showing similarities to our wood and the botanical affinities. The symbols indicate the presence (+), the absence (−) or the lack of information (?) of the anatomical characters. The features in red color can be found both in normal as well as in wood facing biotic or abiotic stress and should be used with caution especially when applied for taxonomic purposes

10.7717/peerj.14212/supp-2 Supplemental Information 2 Comparison of the dimensions of the pith flecks (PF) and the hypertrophic cells (HC) associated with *Paleophytobia* Süss & Müller-Stoll, *Protophytobia* Süss and *Phytobia* Lioy. The available measurements of the pith flecks and the hypertrophic cells from fossil and extant wood with *Palaeo*/*Protophytobia* and *Phytobia* infections are included in this table with the addition of the measurements on our fossil *Cupressinoxylon* with *Protophytobia* from Turkey. The symbol (?) indicates the lack of information.

## Additional Information and Declarations

### Field Study Permissions

The following information was supplied relating to field study approvals (*i.e*., approving body and any reference numbers): The MINISTRY OF AGRICULTURE AND FORESTRY General Directorate of Nature Conservation and National Parks approved this study (E-21264211-288.04-744472). T.C. TARIM VE ORMAN BAKANLIGI Doga Koruma ve Milli Parklar Genel Müdürlügü İstanbul Üniversitesi Cerrahpaşa Orman Fakültesi Orman Botaniği Anabilim Dalı Öğretim Üyesi Prof. Dr. Ünal AKKEMİK tarafından yürütüleceği ifade edilen “Türkiye’nin Jura’dan Pliyosen Dönemine Fosil Ormanlarındaki Ağaç Türlerinin Tespiti ve Koruma Önerilerinin Geliştirilmesi” başlıklı proje kapsamın gerçekleştirilecek olan arazi çalışmaları ile ilgili Bilimsel Araştırma İzin Başvurusu Genel Müdürlüğümüz yetki ve sorumlulukları çerçevesinde incelenmiş olup

### Data Availability

The following information was supplied regarding data availability: The raw data are available in the Supplemental Tables. The material from Gökçeada studied in this work has been permanently accessioned into the recognized collection reposited at Istanbul University-Cerrahpaşa, Forestry Faculty, Department of Forest Botany under the accession number GOK17.
